# β‐catenin‐controlled tubular cell‐derived exosomes play a key role in fibroblast activation via the OPN‐CD44 axis

**DOI:** 10.1002/jev2.12203

**Published:** 2022-03-21

**Authors:** Shuangqin Chen, Meijia Zhang, Jiemei Li, Jiewu Huang, Shan Zhou, Xiaotao Hou, Huiyun Ye, Xi Liu, Shaowei Xiang, Weiwei Shen, Jinhua Miao, Fan Fan Hou, Youhua Liu, Lili Zhou

**Affiliations:** ^1^ State Key Laboratory of Organ Failure Research, National Clinical Research Center of Kidney Disease, Guangdong Provincial Clinical Research Center for Kidney Disease, Guangdong Provincial Key Laboratory of Nephrology, Division of Nephrology Nanfang Hospital Southern Medical University Guangzhou China; ^2^ Division of Nephrology, Ruikang Hospital, Guangxi University of Traditional Chinese Medicine Guangxi Integrated Chinese and Western Medicine Clinical Research Center for Kidney Disease Nanning China; ^3^ Pathology Department Guangzhou KingMed Center for Clinical Laboratory Co., Ltd Guangzhou China; ^4^ Department of Pathology University of Pittsburgh School of Medicine Pittsburgh Pennsylvania USA; ^5^ Bioland Laboratory (Guangzhou Regenerative Medicine and Health, Guangdong Laboratory) Guangzhou China

**Keywords:** CD44, exosomes, OPN, renal fibrosis, β‐catenin

## Abstract

Tubular injury and peripheral fibroblast activation are the hallmarks of chronic kidney disease (CKD), suggesting intimate communication between the two types of cells. However, the underlying mechanisms remain to be determined. Exosomes play a role in shuttling proteins and other materials to recipient cells. In our study, we found that exosomes were aroused by β‐catenin in renal tubular cells. Osteopontin (OPN), especially its N‐terminal fragment (N‐OPN), was encapsulated in β‐catenin‐controlled tubular cell‐derived exosome cargo, and subsequently passed to fibroblasts. Through binding with CD44, exosomal OPN promoted fibroblast proliferation and activation. Gene deletion of β‐catenin in tubular cells (Ksp‐β‐catenin^−/−^) or gene ablation of CD44 (CD44^−/−^) greatly ameliorated renal fibrosis. Notably, N‐OPN was carried by exosome and secreted into the urine of patients with CKD, and negatively correlated with kidney function. The urinary exosomes from patients with CKD greatly accelerated renal fibrosis, which was blocked by CD44 deletion. These results suggest that exosome‐mediated activation of the OPN/CD44 axis plays a key role in renal fibrosis, which is controlled by β‐catenin.

## 1 INTRODUCTION

The patients with chronic kidney disease (CKD) are rapidly increasing and impose a heavy burden for medical care (He et al., 2017; Jha et al., 2012; Tan et al., 2016; Wang et al., 2020). CKD is characterized by the gradual loss of renal function and progression of renal fibrosis (He et al., 2017; Humphreys, 2018), and is a high‐risk factor of end‐stage renal disease (ESRD) (Jha et al., 2012). However, the therapeutic efficacies for CKD remain unsatisfactory nowadays, partly because the underlying mechanisms are poorly understood (Nastase et al., 2018). As the key components of the kidney, renal tubular epithelial cells and fibroblasts are major participants in renal fibrosis (Kang et al., 2015; Liu, 2011; Liu et al., 2020; Zhou & Liu, 2016), and recent findings have shown that the communication exists between them (Gewin et al., 2017; Liu et al., 2020; Prunotto et al., 2012; Qi & Yang, 2018; Tan et al., 2016). The message delivery may be mediated by a functional protein and gene loader‐exosome (Guan et al., 2020; Wortzel et al., 2019).

Exosomes are the lipid bilayer vesicles with sizes ranging between 30 and 150 nm (He et al., 2018). Through secretion and endocytosis, exosomes mediate neighboring cell crosstalk and even trigger the communication to remote organs (Wortzel et al., 2019). Carrying various biomolecules, such as proteins, lipids, metabolites, glycans and RNAs, exosomes can be excreted into blood, urine and even breast milk (He et al., 2018; Wortzel et al., 2019). Exosome‐mediated tubule‐interstitial communication was recently shown to be a main mechanism underlying the development of renal fibrosis (Guan et al., 2020; Liu et al., 2020). Nevertheless, the detailed mechanisms remain to be elucidated.

As the main constituent of the renal parenchyma, tubular epithelial cells are vulnerable to undergo dedifferentiation, epithelial‐mesenchymal transition (EMT), apoptosis, cell cycle arrest and cellular senescence (Grande et al., 2015; Xu et al., 2020; Yang et al., 2010; Zhou & Liu, 2016). In addition to the morphological changes, tubular epithelial cells commonly acquire a secreted phenotype to release proinflammatory and profibrotic factors, including IL‐1β, IL‐6, IL‐8, CXCL1 and TGF‐β1, all of which are drivers to renal fibrosis (Qi & Yang, 2018; Xu et al., 2020). Interestingly, as a type of cell with active metabolism, renal tubular epithelial cells frequently undertake endocytosis and exocytosis. Exosome cargo‐mediated message transmission could certainly play a central role between tubular cells and neighboring cells such as fibroblasts. Indeed, our recent finding showed that tubule‐derived exosomes induce fibroblast activation and lead to renal fibrosis through exosome‐encapsulated sonic hedgehog (Liu et al., 2020). However, the components in tubular cell‐derived exosomes and its mediative role in tubular cell and interstitial fibroblast communication have not been fully elucidated.

Osteopontin (OPN) is an extracellular matrix glyco‐phosphoprotein, which was first identified in bone tissue and then found to be expressed in other tissues, such as kidney, tooth and blood (Subraman et al., 2015; Vianello et al., 2020). Though binding with its receptor CD44 and integrin β3, OPN mediates cell adhesion, proliferation, invasion and apoptosis, and plays a role during tissue fibrosis (Bai et al., 2020; Kaleta, 2019). Full‐length OPN has a molecular weight of approximately 70 kDa. In the diseased state, full‐length OPN is often cleaved into an N‐terminal fragment (∼50 kDa) and two C‐terminal fragments (∼18 and ∼16 kDa) (Hoac et al., 2018). N‐OPN could be the major active form to trigger a series of pathological changes (Hattori et al., 2021; Liu et al., 2021). However, the role of N‐OPN in kidney injury has not been investigated yet. Furthermore, the underlying mechanisms and modulating signals are poorly understood.

In this study, we found that OPN, especially N‐OPN, was increased in injured tubules and secreted into the urine by exosomal capsulation. Urinary N‐OPN served as an indicative marker for renal fibrosis and decline of kidney function. OPN‐encapsulated exosomes mediate intercellular message transmission from injured tubular cells to interstitial fibroblasts, through the receptor of CD44. We further identified that OPN is a new downstream target of β‐catenin, a master controller in tubular injury and fibrogenesis. This study demonstrates that exosome‐mediated activation of the OPN/CD44 axis plays a key role in renal fibrosis, which is controlled by β‐catenin. Our findings provide a new mechanism of renal fibrosis and implicate an important therapeutic strategy to CKD.

## 2 RESULTS

### 2.1 N‐OPN increases in kidney and urine in various clinical nephropathies and associates with CKD progression primarily through encapsulation within exosomes

**FIGURE 1:**
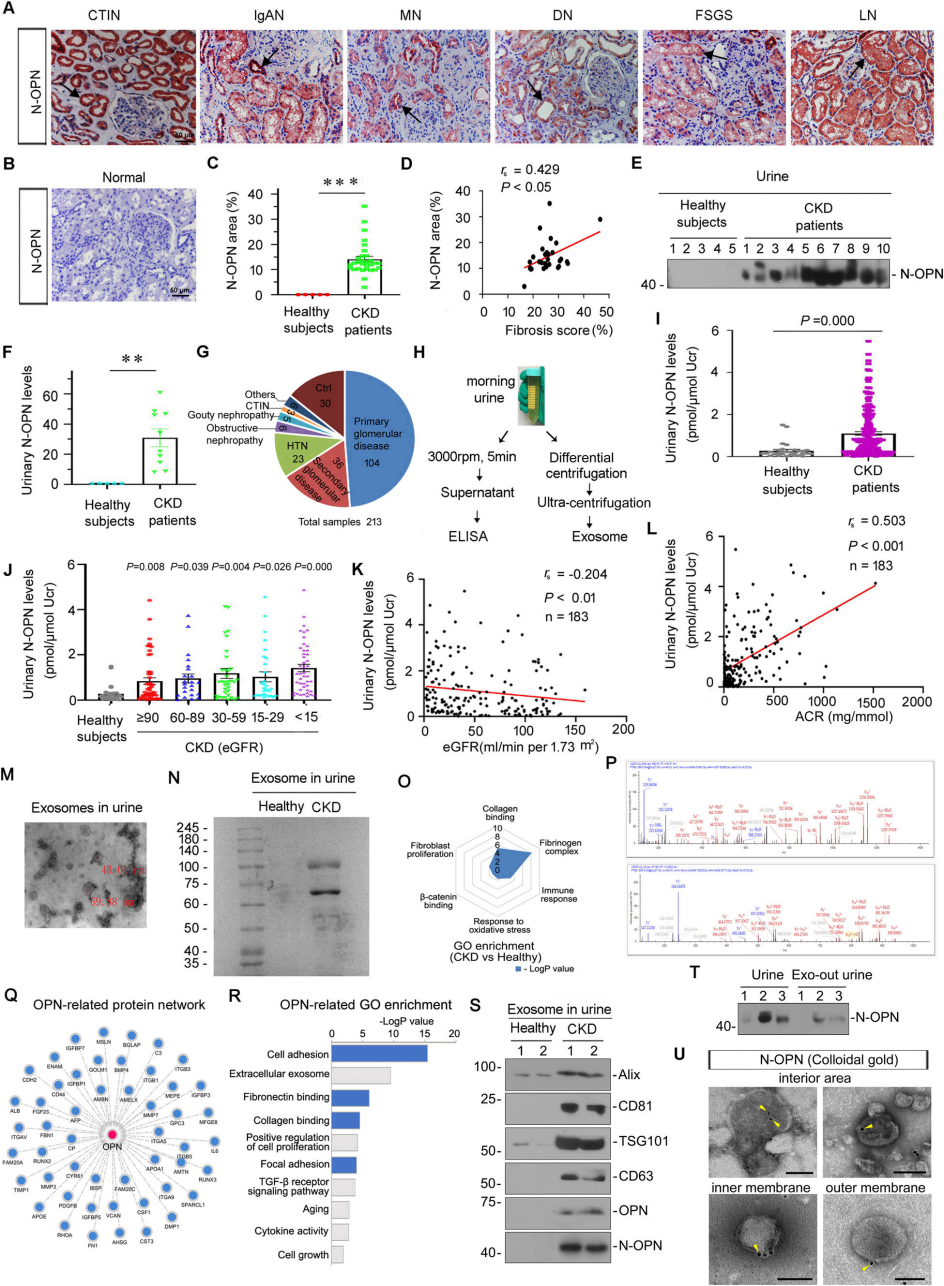
N‐OPN increases in kidney and urine in various clinical nephropathies and associates with CKD progression primarily through encapsulation within exosomes. (a) Representative micrographs showing the abundance and localization of N‐OPN protein in various human CKD: chronic tubulo‐interstitial nephritis (CTIN), IgA nephropathy (IgAN), membranous nephritis (MN), diabetic nephropathy (DN), focal segmental glomerulosclerosis (FSGS) and lupus nephritis (LN). (b) Normal: nontumor kidney tissue from patients with renal cell carcinoma were used as healthy subjects. Arrow indicate positive staining (scale bar: 50 μm). (c) Quantitative analysis of immunohistochemical staining of N‐OPN in patients with CKD and healthy subjects. (d) Correlation between N‐OPN and fibrosis score. (e–f) Western blot analyses show urinary N‐OPN protein in healthy subjects and patients with CKD. Representative western blot (e) and quantitative data (f) are shown. Numbers (1–10) indicate urine samples from each subject. **p < 0.01 versus healthy subjects. (g) Pie chart shows the composition of human urine samples. (h) Diagram shows the experimental plan. Morning urine was collected, centrifuged at 3000 rpm for 5 min. The supernatant was collected and tested by ELISA; in addition, after differential centrifugation, the exosomes were extracted by ultracentrifugation. (i) Graphic presentation shows urinary N‐OPN protein levels in cohorts of patients with CKD (*n* = 183) and healthy subjects (*n* = 30). Urinary N‐OPN levels are presented as pmol/μmol urinary creatinine (Ucr). ***p < 0.001 versus healthy subjects. (j) Graphic presentation shows urinary N‐OPN protein levels in different stages of CKD. There was no statistical difference among different CKD stages. (k) Linear regression shows a negative correlation between urinary N‐OPN protein and kidney function (estimated glomerular filtration rate [eGFR]). (l) Linear regression shows a significant correlation between urinary N‐OPN levels and urinary albumin to creatinine ratio (ACR). (m–t) Analyses of exosomes isolated from the urine of patients with CKD and healthy subjects. (m) Transmission electron microscopy (TEM) image showing the exosomes isolated from urine from patients with CKD. (n) Representative images of Coomassie blue staining of exosomes from the urine of healthy subjects and patients with CKD. (o) Gene ontology (GO) enrichment analysis of the specific proteins from urinary exosomes of patients with CKD. (p) Representative micrographs showing two specific peptides of OPN from urinary exosomes isolated from CKD patients were identified by mass spectrometry. (q) An interaction network of OPN protein with other proteins was identified using the STRING database. (r) GO enrichment analysis of OPN‐interacted proteins shows the potential function of OPN. (s) Exosomes were prepared from the same amounts of urine from healthy subjects or patients with CKD, and were lysed and immunoblotted with antibodies against Alix, CD81, TSG101, CD63, OPN and N‐OPN, respectively. (t) Western blot analyses show N‐OPN protein expression in urine and exosome‐removed urine from patients with CKD. (u) Colloidal gold electron microscopy analysis demonstrates that N‐OPN was encapsulated in urinary exosomes from patients with CKD. N‐OPN was labelled with 10 nm colloidal gold particles. Arrows indicate positive staining; Scale bar: 100 nm

We first examined the expression of N‐OPN in various clinical nephropathies. As shown in Figure 1a–c, compared to the negative signal in normal human kidney, N‐OPN was induced in kidney biopsies from patients with CKD, including chronic tubulo‐interstitial nephritis, IgA nephropathy, membranous nephritis, diabetic nephropathy, focal segmental glomerulosclerosis and lupus nephritis. N‐OPN protein was predominantly localized in tubules in diseased kidneys, but less expressed in the interstitium and glomeruli. We further assessed fibrotic lesions and found that there was a positive correlation between N‐OPN expression and renal fibrosis (Figure 1d).

As N‐OPN is a secreted protein, we further assessed its expression in the urine from patients with CKD by western blotting analyses. As shown in Figure 1e and f, N‐OPN was greatly elevated in the urine of patients with CKD, but not in healthy subjects. To test the significance of urinary N‐OPN, we further quantitatively measured the levels in 30 healthy subjects and a cohort of 183 patients with CKD using a specific ELISA. The demographic and clinical data of the patients are presented in Figure 1g and Table S1. Some urine samples were also centrifuged to generate exosomes (Figure 1h). Notably, urinary N‐OPN levels were significantly elevated in patients with CKD compared to those healthy subjects (Figure 1i and j). Notably, as shown in Figure 1k and l, N‐OPN levels were inversely correlated with the estimated glomerular filtration rate, and positively correlated with urinary albumin secretion. We then isolated exosomes from the urine of CKD patients (Figure 1 m) and tested its protein constitution by SDS–PAGE with Coomassie Blue staining (Figure 1n) and mass spectrometry. As shown in Figure 1o–r, gene ontology enrichment analysis of the differentially expressing proteins revealed that the urinary exosomes from patients with CKD significantly expressed the proteins involved in the fibrinogen complex, collagen binding, immune response, fibroblast proliferation, response to oxidative stress and β‐catenin binding pathway, compared with the healthy controls (Figure 1o). Interestingly, the specific peptides of OPN protein were identified in urinary exosomes from patients with CKD (Figure 1p). To explore the function of OPN, the STRING database was used to predict OPN‐interacting proteins (Figure 1q), which were then subjected to GO enrichment analysis (Figure 1r). Gene ontology analysis showed that OPN‐related pathways were involved in cell adhesion, extracellular exosomes, fibronectin binding, collagen binding, cell proliferation, and other processes involved in fibrogenesis signalling, such as focal adhesion, TGF‐β and aging. As a note, the majority of pathways involved in OPN functions are similar to those found in the encapsulated proteins in the urinary exosomes from patients with CKD. This finding suggests that OPN would play an important role in CKD progression through encapsulation in exosomes. To verify this hypothesis, urinary exosomes were assessed by western blotting analyses. As shown in Figure 1s and Figure S1A–F, exosome markers Alix, CD81, TSG101 and CD63 (Lv et al., 2020; Tang et al., 2020), and OPN and N‐OPN were significantly upregulated in urinary exosomes from patients with CKD. To further confirm the ratio of exosomal N‐OPN in whole urine, we assessed the expression of N‐OPN in the whole urine of CKD patients and the urine with exosomes removed. Interestingly, after exosomes were removed, the expression of N‐OPN was extremely weak in the urine. This indicates that N‐OPN is primarily expressed (approximately over 70%) in urinary exosomes (Figure 1t and Figure S1G). Furthermore, immunocolloidal gold staining verified that N‐OPN is indeed encapsulated in the urinary exosomes isolated from patients with CKD, and it is in the interior area, and also at the inner and outer membranes of exosomes (Figure 1u). These results showed that N‐OPN was secreted via exosomes into the urine of patients with CKD, and served as an indicative marker for CKD progression and renal fibrosis. The identification of exosomes (exosome quantification, gel analysis of silver staining, iodixanol density gradient centrifugation and nanoparticle tracking analysis (NTA)) are shown in Figure S1H–K.

### 2.2 OPN and N‐OPN are upregulated in various models of CKD and encapsulated within tubular exosomes

**FIGURE 2:**
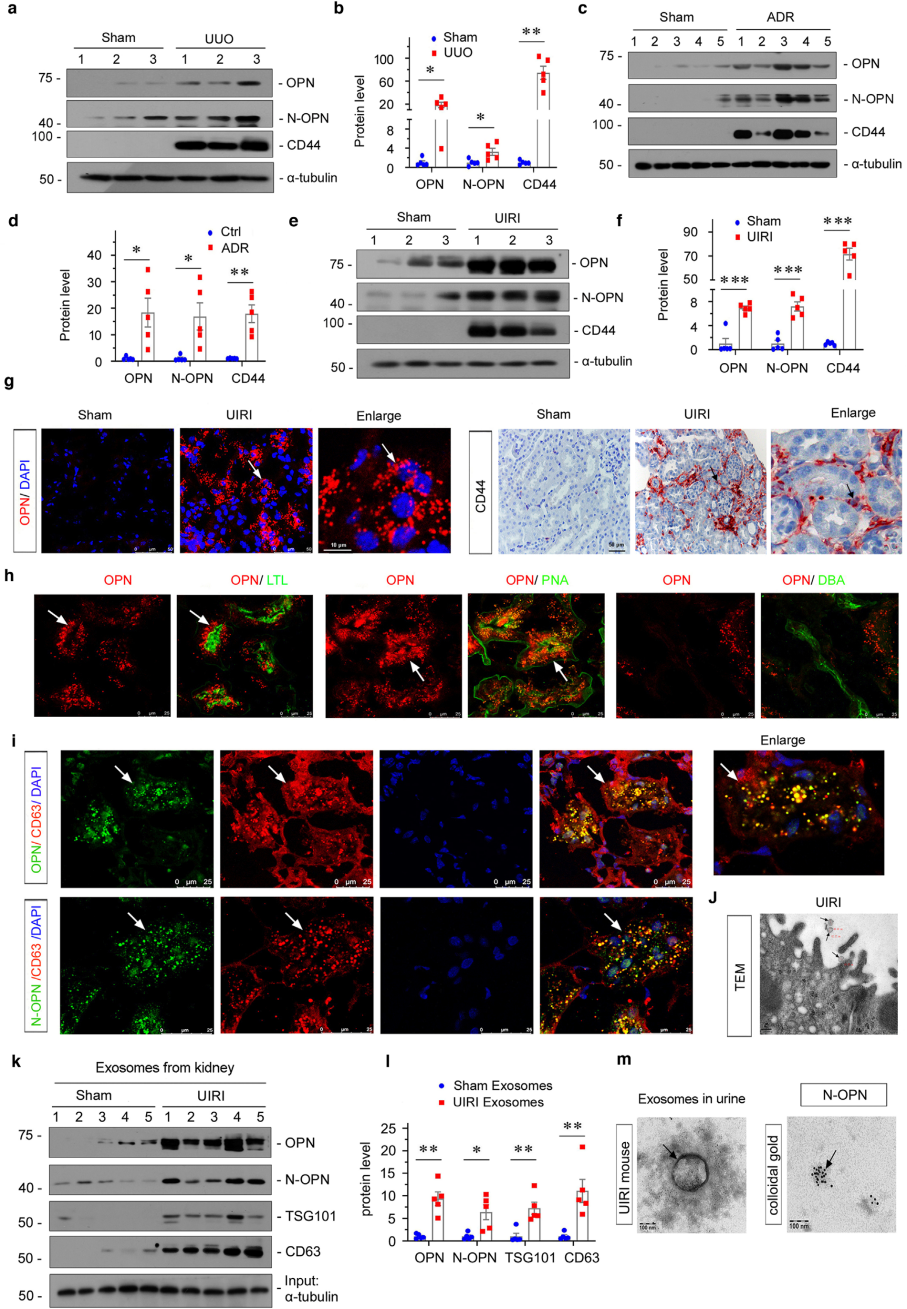
OPN and N‐OPN are upregulated in various models of CKD and encapsulated within tubular exosomes. (a and b) Representative western blots (a) and quantitative data (b) show the induction of renal OPN, N‐OPN and CD44 expression after UUO. Numbers (1 to 3) indicate individual animals in a given group. Relative OPN, N‐OPN and CD44 levels are expressed as fold induction over sham controls after normalization with α‐tubulin. *p < 0.05, **p < 0.01 versus controls (*n* = 5). (c and d) Western blotting analyses demonstrate renal expression of OPN, N‐OPN and CD44 after injection of Adriamycin (ADR). Numbers (1 to 5) indicate individual animals in a given group. *p < 0.05, **p < 0.01 versus controls (*n* = 5). (e and f) Western blot analyses show renal expression of OPN, N‐OPN and CD44 protein after UIRI. Numbers (1 to 3) indicate individual animals in a given group. ***p < 0.001 versus controls (*n* = 5). (g) Representative micrographs of immunofluorescence and immunohistochemical staining show tubular OPN and interstitial CD44 expression in UIRI mice. Arrow indicates positive staining; scale bar: 50 μm. (h) Double immunofluorescence staining demonstrates the generation of OPN predominantly in the proximal and distal tubular epithelium. Kidney sections were co‐stained for OPN (red) and various segment‐specific tubular markers (green). Segment‐specific tubular markers were used as follows: proximal tubule, lotus tetragonolobus lectin (LTL); distal tubule, peanut agglutinin (PNA); and Dolichos biflflorus agglutinin (DBA). Arrow indicates positive staining; scale bar: 25 μm. (i) Double immunofluorescence staining demonstrates that OPN and N‐OPN co‐localize with CD63. Kidney sections were co‐stained for CD63 (red) and OPN or N‐OPN (green). Boxed area is enlarged; Arrow indicates positive staining; scale bar: 25 μm. (j) TEM showing the exosomes released in kidney tissue after UIRI. Arrows indicate exosomes; scale bar: 200 nm. (k and l) Western blot analyses show the presence of OPN, N‐OPN, TSG101 and CD63 proteins in the exosomes isolated from kidneys after UIRI. Exosomes were isolated form kidney tissue of the same weight. Representative western blot (k) and quantitative data (l) are shown. Numbers (1–5) indicate a pool of exosomes isolated from one animal. *p < 0.05, **p < 0.01 versus controls (*n* = 5). (m) TEM show the exosome isolated from urine of UIRI mice and colloidal gold staining demonstrates that N‐OPN was encapsulated by them. N‐OPN was labelled with 10 nm colloidal gold. Arrow indicates N‐OPN; scale bar: 100 nm

To test the expression of both full‐length OPN (∼70 kDa) and active, N‐terminal OPN (N‐OPN, ∼50 kDa) in CKD, we constructed various CKD mouse models of unilateral ureteral obstruction (UUO), adriamycin nephropathy (ADR) and unilateral ischemia/reperfusion injury (UIRI). As shown in Figure 2a–f, the expressional levels of OPN, and N‐OPN were significantly increased in kidneys after UUO, ADR or UIRI. Interestingly, CD44, the receptor of OPN (Klement et al., 2018), was also induced.

We next assessed the location of OPN and CD44 in the kidneys by immunofluorescence or immunohistochemistry staining. As shown in Figure 2g, in UIRI mice, there was substantial expression of OPN and CD44 compared with the weak signal in sham kidneys. Interestingly, OPN was primarily expressed in tubular epithelial cells, while CD44 was mainly located in the interstitial area. To further clarify the expression of OPN in tubules, we performed co‐staining for OPN and different segment‐specific tubular cell markers, including lotus tetragonolobus lectin, a marker of proximal tubules, peanut agglutinin, a maker of distal tubules and Dolichos biflflorus agglutinin, a marker of the collecting duct. As shown in Figure 2h, proximal, and distal tubular epithelial cells were identified as the major sources of OPN, whereas OPN was barely detectable in the collecting duct. We next performed co‐staining of OPN and CD63, an exosome marker. As shown in Figure 2i, the co‐localization of CD63 and OPN was confirmed, suggesting that OPN was encapsulated in exosomes. A similar result was observed when N‐OPN and CD63 were co‐stained. The exosomes derived from proximal tubular epithelial cells were also observed by transmission electron microscopy (TEM) (Figure 2j). Exosomes were also isolated from the kidneys and urine in mice after UIRI. As shown in Figure 2k and l, the exosomes from UIRI‐affected kidneys showed high expression of OPN, N‐OPN, TSG101 and CD63. To further confirm that N‐OPN is secreted through encapsulation in exosomes, we isolated exosomes from the urine of UIRI mice and then performed immunocolloidal gold staining. As shown in Figure 2m, N‐OPN was indeed secreted into urine within exosomes.

### 2.3 Ectopic OPN promotes renal fibrosis, which is inhibited by the prevention of exosome secretion in vivo

**FIGURE 3:**
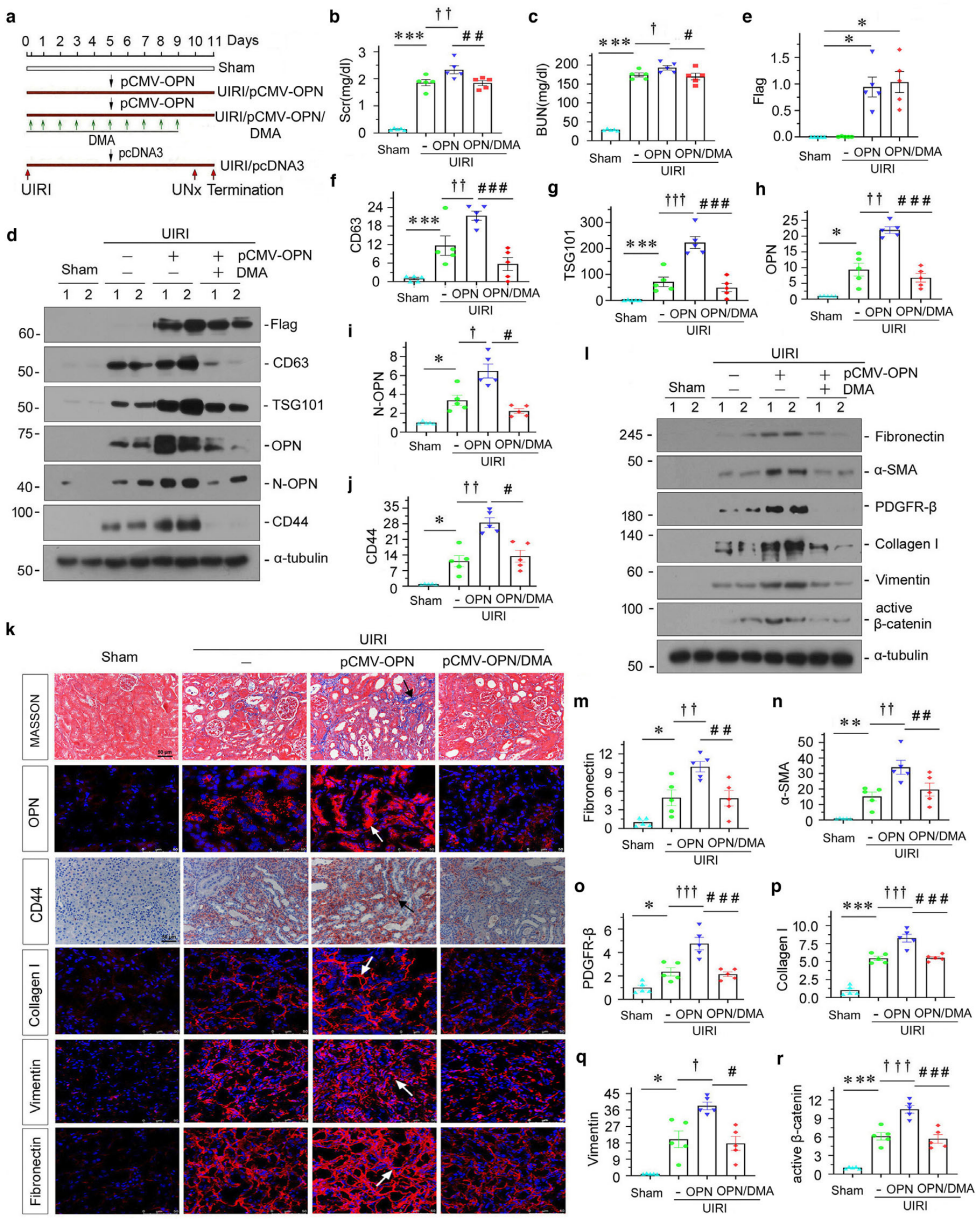
Overexpression of OPN promotes renal fibrosis but blocking with exosome inhibitor dimethyl amiloride (DMA) inhibits kidney fibrosis in vivo. (a) Diagram showing the experimental design. Black arrows indicate the injection of pcDNA3 or OPN overexpression plasmid (pCMV‐OPN). Red arrows indicate the time points undergoing UIRI, UNx and sacrifice. Green arrows indicate dimethyl amiloride (DMA) treatment (10 mg/kg body weight). (b and c) Graphic presentation showing the serum creatinine levels (b) and BUN levels (c) in different groups after UIRI. ***p < 0.001 versus sham controls; ^†^ *p* < 0.05, ^††^ *p *< 0.01 versus UIRI (*n* = 5); ^#^ *p *< 0.05, ^##^ *p *< 0.01 versus UIRI + pCMV‐OPN (*n* = 5). Western blot (d) and quantitative data (e–j) showing the upregulation of renal Flag, CD63, TSG101, OPN, N‐OPN and CD44 expression after overexpression OPN, but these effects were abolished after DMA treatment. *p < 0.05, ***p < 0.001 versus sham controls; ^†^ *p *< 0.05, ^††^ *p *< 0.01, ^†††^ *p* < 0.001 versus UIRI; ^#^ *p *< 0.05, ^###^ *p *< 0.001 versus UIRI + pCMV‐OPN (*n* = 5). (k) Kidney tissues from different groups were subjected to Masson's trichrome staining, immunofluorescence staining of OPN, collagen I, vimentin and fibronectin, and immunostaining staining for CD44, as indicated respectively. Arrows indicate positive staining; scale bar: 50 μm. (l–r) Western blots analyses show renal expression of fibrosis‐related proteins. Representative western blot (l) and quantitative data for fibronectin (m), α‐SMA (n), PDGFR‐β (o), collagen I (p), vimentin (q) and active β‐catenin (r) proteins are shown. Numbers (1 to 2) indicate individual animal in a given group. *p < 0.05, **p < 0.01, ***p < 0.001 versus sham controls; ^†^ *p *< 0.05, ^††^ *p *< 0.01, ^†††^ *p* < 0.001 versus UIRI; ^#^ *p *< 0.05, ^##^ *p *< 0.01, ^###^ *p *< 0.001 versus UIRI + pCMV‐OPN (*n* = 5)

To clarify the role of exosomal OPN in renal fibrosis, we performed experiments in UIRI mice by intravenous injection of OPN expression vector (pCMV‐OPN) and concomitantly treated mice with DMA to block the release of exosomes. The experimental design is presented in Figure 3a. We then examined kidney function. As shown in Figure 3b and c, serum creatinine (Scr) and blood urea nitrogen (BUN) levels were significantly elevated after UIRI, but the overexpression of OPN further augmented them. However, cotreatment with DMA inhibited these effects. The efficacy of OPN overexpression was confirmed by western blotting (Figure 3d and e). CD63, TSG101, OPN, N‐OPN and CD44 expressional levels were increased after the overexpression of OPN in UIRI mice, but were significantly blocked by the administration of DMA (Figure 3d–j). A similar result was observed when OPN and CD44 were assessed by immunofluorescence or immunohistochemistry staining (Figure 3k). To further confirm the role of OPN in CKD, we then assessed renal interstitial fibrotic lesions by Masson's trichrome staining (Masson) and tested the expression of multiple fibrogenesis‐related proteins by western blotting. Overexpression of OPN deteriorated renal fibrosis, characterized by increased deposition of collagen (Figure 3k). Furthermore, as shown in Figure 3l–r, the expressional levels of fibronectin, α‐SMA, PDGFR‐β, collagen I, vimentin and active β‐catenin were all upregulated after UIRI injury, and further exacerbated by overexpression of OPN, but were largely blocked by co‐treatment with DMA. Similar results were observed when collagen I, vimentin and fibronectin were examined by immunofluorescence staining (Figure 3k).

**FIGURE 4:**
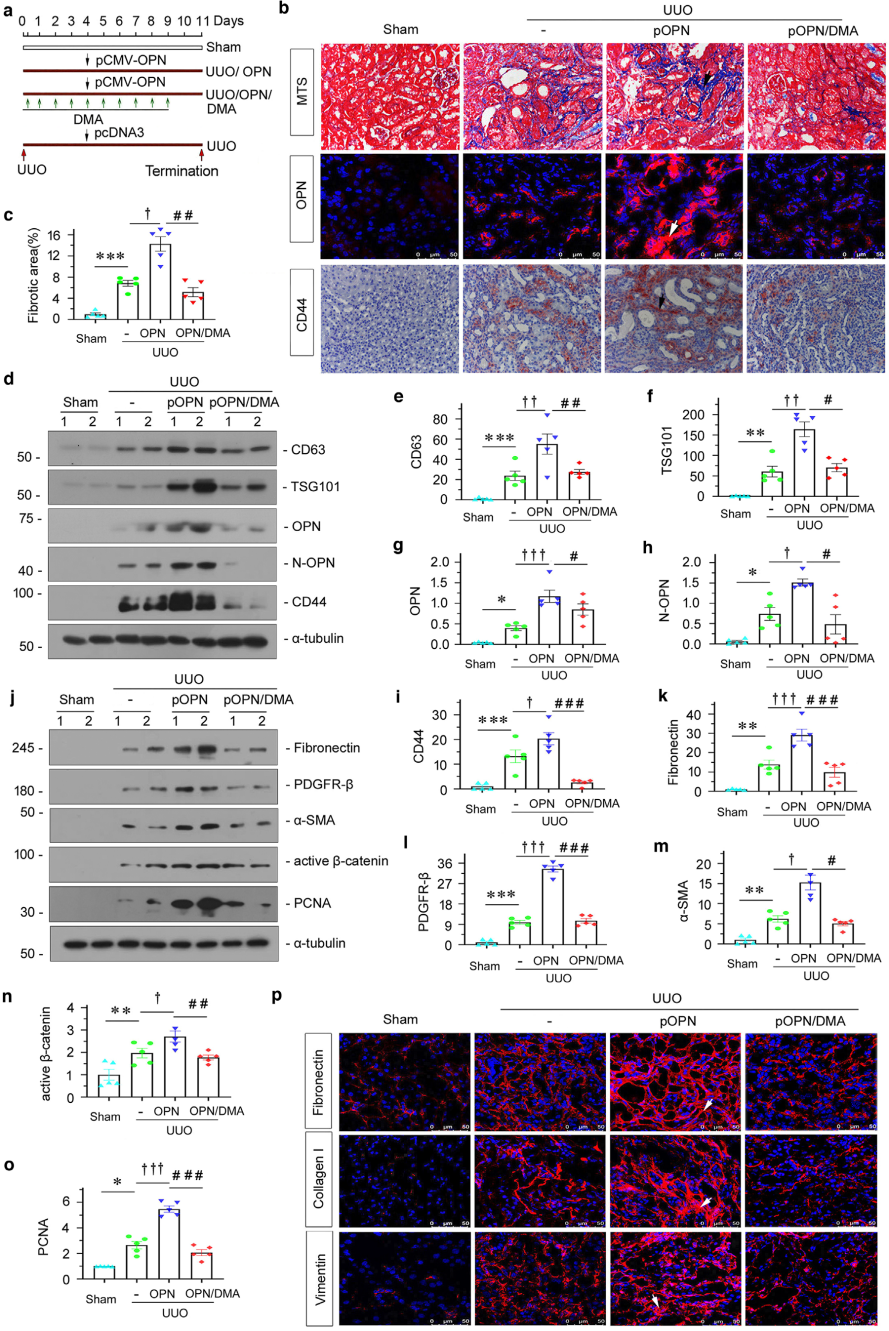
Ectopic OPN promotes renal fibrosis, which is inhibited by the prevention of exosome secretion in a UUO model. (a) Experimental design. Red arrows indicate the time points undergoing UUO and sacrifice. Green arrows indicate dimethyl amiloride (DMA) treatment (10 mg/kg body weight). Black arrow indicates the injection of pcDNA3 or OPN overexpression plasmid (pMCV‐OPN). (b) Representative micrographs showing collagen deposition, OPN and CD44 expression in different groups, as indicated. Kidney sections were subjected to Masson's trichrome staining for collagen deposition (upper), immunofluorescence staining of OPN (middle) and immunohistochemical staining of CD44 (bottom) in different groups, as indicated. Arrow indicates positive staining; scale bar: 50 μm. (c) Graphic presentation showing the quantitative determination of kidney fibrotic lesions in different groups. ***p < 0.001 versus sham controls; ^†^ *p *< 0.05 versus UUO; ^##^ *p *< 0.01 versus UUO + pCMV‐OPN (*n* = 5). (d–i) Western blot analyses showing protein expression of CD63, TGS101, OPN, N‐OPN and CD44 in different groups, as indicated. Representative western blot (d) and quantitative data for CD63 (e), TSG101 (f), OPN (g), N‐OPN (h) and CD44 (i) are shown. Numbers (1 to 2) indicate individual animal in a given group. *p < 0.05, **p < 0.01, ***p < 0.001 versus sham controls; ^†^ *p *< 0.05, ^††^ *p *< 0.01, ^†††^ *p *< 0.001 versus UUO; ^#^ *p *< 0.05, ^##^ *p *< 0.01, ^###^ *p *< 0.001 versus UUO + pCMV‐OPN (*n* = 5). (j–o) Western blot analyses showing fibrosis‐related protein expression of fibronectin, PDGFR‐β, α‐SMA, active β‐catenin and PCNA in different groups, as indicated. Representative western blot (j) and quantitative data for fibronectin (k), PDGFR‐β (l), α‐SMA (m), active β‐catenin (n) and PCNA (o) are shown. *p < 0.05, **p < 0.01, ***p < 0.001 versus sham controls; ^†^ *p *< 0.05, ^†††^ *p *< 0.001 versus UUO; ^#^ *p *< 0.05, ^##^ *p *< 0.05, ^###^ *p *< 0.001 versus UUO + pCMV‐OPN (*n* = 5). (p) Representative micrographs showing immunofluorescence staining of fibronectin, collagen I and vimentin in different groups, as indicated. Arrows indicate positive staining; scale bar: 50 μm

To further confirm the role of exosomal OPN in promoting fibrosis, we established a UUO mouse model (Figure 4a). In accordance with UIRI, OPN significantly triggered the deposition of collagen and fibronectin. However, co‐treatment with DMA decreased the deposition of collagen and fibronectin, compared with UUO mice with OPN treatment alone (Figure 4b and c), as assessed by Masson staining. Similarly, the efficacy of OPN overexpression was confirmed by western blotting (Figure S5K–L). Then, we further assessed the expression of CD63, TSG101, OPN, N‐OPN and CD44 protein by western blotting, immunofluorescence and immunohistochemical staining. As shown in Figure 4b and d–i, CD63, TSG101, N‐OPN, OPN and CD44 expression levels were elevated by overexpression of OPN, but declined following co‐treatment with DMA. To further confirm the role of OPN in promoting fibroblast activation and proliferation, we then tested fibrotic lesions and fibroblast proliferation by western blotting and immunofluorescence. As shown in Figure 4j–p, OPN induced the upregulation of fibronectin, PDGFR‐β, α ‐SMA, active β‐catenin, PCNA, collagen I and vimentin, but these effects were attenuated by cotreatment with DMA. Although the profibrotic effects of OPN on fibroblasts were confirmed in cultured fibroblasts in vitro (Figure S3), ectopic expression of OPN or injection with DMA in normal mice did not affect fibrogenesis (Figure S2). This further suggests that exosomal OPN may play a central role in mediating the pathological processes in renal fibrosis.

### 2.4 β‐catenin‐controlled tubular cell‐derived exosomes mediate fibroblast activation in vitro

**FIGURE 5:**
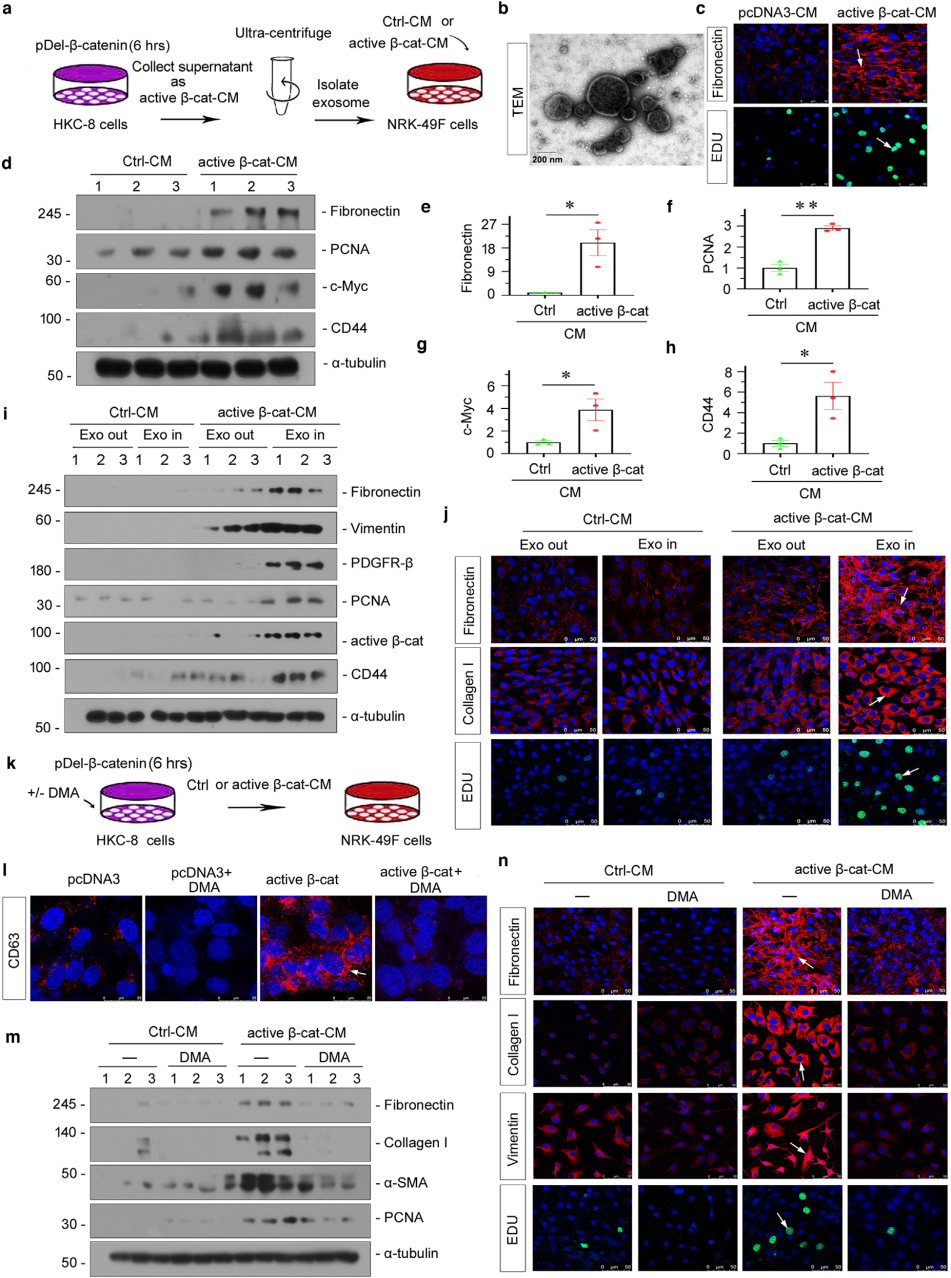
Tubular cell‐derived exosomes controlled by β‐catenin promote fibroblast activation in vitro. (a) Experimental design. Human kidney proximal tubular epithelial cells (HKC‐8) were transfected with active β‐catenin expression plasmid (pDel‐β‐catenin) for 6 h and then washed and continued to be incubated for an additional 24 h in serum‐free medium (active β‐cat conditioned medium). Exosomes were isolated from conditioned media by ultracentrifugation. Conditioned media from HKC‐8 cells were collected and used to stimulate normal rat kidney interstitial fibroblasts (NRK‐49F). (b) TEM showing the exosomes isolated from the conditioned media of HKC‐8 cells; scale bar: 200 nm. (c–j) Conditioned media from HKC‐8 cells that were transfected with active β‐catenin plasmid could stimulate the activation in NRK‐49F cells in vitro. NRK‐49F cells were treated with 40% of active β‐cat conditioned medium for 24 h. (c) Representative micrographs showing immunofluorescence staining of fibronectin and EDU staining in NRK‐49F cells after incubation with conditioned media from HKC‐8 cells. Arrow indicates positive staining; scale bar: 50 μm. (d–h) Representative western blot (d) and quantitative data (e–h) show that the expression of fibronectin (e), PCNA (f), c‐Myc (g) and CD44 (h) was induced in NRK‐49F cells by conditioned media from HKC‐8 cells. *p < 0.05, **p < 0.01 versus pcDNA3‐CM; numbers (1 to 3) indicate individual treatment in a given group. (i, j) Exosomes are necessary to mediate the activation in fibroblasts induced by conditioned media from HKC‐8 cell. (i) Western blot analyses show that the conditioned media lacking exosomes from HKC‐8 cells failed to induce fibronectin, vimentin, PDGFR‐β, PCNA, active β‐catenin and CD44 expression in NRK‐49F cells. (j) Representative micrographs showing immunofluorescence staining of fibronectin (upper), collagen I (middle), and EDU staining (bottom) in different groups, as indicated. Arrow indicates positive staining; scale bar: 50 μm. (k) HKC‐8 cells were transfected with pDel‐β‐catenin for 6 h in the absence or presence of dimethyl amiloride (DMA),and continued to be incubated for additional 24 h in serum‐free medium. Conditioned media were collected and used to stimulate NRK‐49F cells. HKC‐8 cells were pretreated with 100 μmol/L of DMA for 1 h, and transfected with pDel‐β‐catenin plasmid for 6 h, and then incubated with serum‐free medium for 24 h (active β‐cat conditioned medium). NRK‐49F cells were treated with 40% of active β‐cat conditioned medium for 24 h. (l) Immunofluorescence staining of CD63 shows that DMA, an exosome inhibitor, inhibited exosome secretion. Representative immunofluorescence staining micrographs of CD63 are shown, as indicated. Arrow indicates positive staining; scale bar: 50 μm. (m) Western blot analyses show that the blockade of exosome generation by DMA inhibited the expression of fibronectin, collagen I, α‐SMA and PCNA in NRK‐49F cells induced by conditioned media from HKC‐8. (n) Representative micrographs of immunofluorescence staining of fibronectin, collagen I, vimentin and EDU in NRK‐49F cells after incubation with conditioned media, as indicated. Arrow indicates positive staining; scale bar: 50 μm

β‐catenin is a master controller for renal tubular cell injury by initiating multiple downstream targets, such as RAAS, PAI and MMP‐7. Hence, we further identified the role of β‐catenin in controlling tubule‐derived exosomal OPN. We first isolated exosomes from the conditioned medium of cultured human renal tubular epithelial cells (HKC‐8) transfected with active β‐catenin expression plasmid (pDel‐β‐catenin), and then treated fibroblasts (NRK‐49F) (Figure 5a). As shown in Figure 5b and Figure S6A, TEM and NTA showed that exosomes were successfully isolated, as confirmed by their sizes and bilayer membrane structure. Furthermore, conditioned medium from active‐β‐catenin‐overexpressed HKC‐8 cells (active‐β‐catenin‐CM) could induce cell proliferation and activation in NRK‐49F cells, as manifested by increased expression of fibronectin, EDU‐positive cells, PCNA and c‐Myc (Figure 5c–g). We also observed an increase in CD44 expression (Figure 5d and h), suggesting the production of OPN in β‐catenin‐overexpressed HKC‐8 cells.

To discern the role of exosomes in mediating tubule‐fibroblast communication, we treated NRK‐49F cells with complete conditioned media (Exo In) or exosome‐depleted conditioned media (Exo Out). We found that complete active‐β‐catenin‐CM (Exo In) induced fibronectin, vimentin, PDGFR‐β, PCNA, active β‐catenin and CD44 upregulation in NRK‐49F cells. However, depletion of exosomes from active β‐catenin‐CM (Exo Out) abolished the induction of these proteins (Figure 5i and Figure S4A–F). Consistently, fibronectin, collagen I and EDU staining showed that they were induced by active‐β‐catenin‐CM (Exo In), but were abolished when exosomes were deprived (Exo Out) (Figure 5j).

To further study the role of exosomes in mediating tubule‐fibroblast communication, we transfected HKC‐8 cells with pDel‐β‐catenin or pcDNA3 plasmid. After transfection (6 h), HKC‐8 cells were incubated with dimethyl amiloride (DMA), a blocker to exosome release. After 24 h, we collected the conditioned medium to stimulate NRK‐49F cells (Figure 5k). The inhibition of DMA on exosome secretion was confirmed by CD63 immunofluorescence staining (Figure 5l). As shown in Figure 5 m and n, and Figure S4G–J, co‐treatment with DMA inhibited the expression of fibronectin, collagen I, α‐SMA and PCNA. Similarly, blockade of exosome secretion by DMA also abolished cell proliferation and activation in cultured fibroblasts, as demonstrated by vimentin and EDU staining (Figure 5n).

### 2.5 Exosome‐mediated OPN/CD44 axis in tubule‐fibroblast communication is controlled by β‐catenin

**FIGURE 6:**
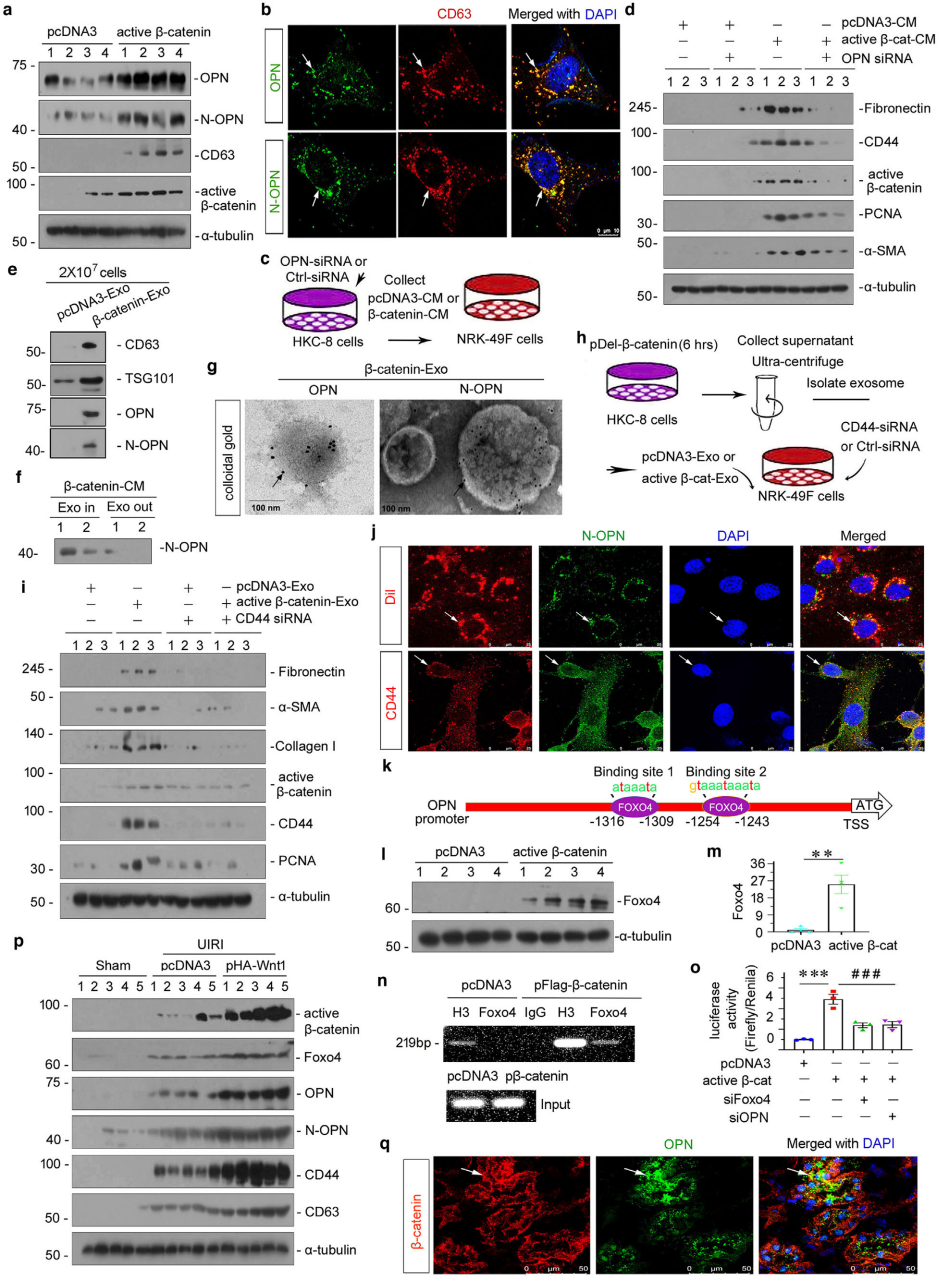
Exosome‐mediated activation of the OPN/CD44 axis in tubule‐fibroblast communication is controlled by β‐catenin. (a) Western blot analyses showing protein expression of OPN, N‐OPN, CD63 and active β‐catenin in Human kidney proximal tubular (HKC)‐8 cells transfected with pDel‐β‐catenin for 24 h. Numbers (1 to 4) indicate individual treatment in a given group. (b) Colocalization of CD63 and OPN (upper), and CD63 and N‐OPN (bottom) is shown by double immunofluorescence staining in HKC‐8 cells transfected with pDel‐β‐catenin for 24 h. Arrow indicates positive staining; scale bar: 10 μm. (c) Experimental design shows OPN was knocked down in HKC‐8 cells prior to the collection of conditioned media. HKC‐8 cells were cotransfected with siRNA to OPN and pDel‐β‐catenin plasmid for 6 h, and then incubated with serum‐free medium for 24 h (active β‐cat conditioned medium). NRK‐49F cells were treated with 40% of conditioned medium for 24 h. (d) Western blot shows that knockdown of OPN in HKC‐8 cells abolished the expression of fibronectin, CD44, active‐β‐catenin, PCNA and α‐SMA in NRK‐49F cells after incubation with conditioned media. Numbers (1 to 3) indicate individual treatment in a given group. (e) Western blot analyses demonstrate the presence of CD63, TSG101, OPN and N‐OPN proteins in the exosomes isolated from HKC‐8 cells transfected with pDel‐β‐catenin. Exosomes prepared from the same amounts of HKC‐8 cells transfected with pDel‐β‐catenin were lysed and immunoblotted with antibodies against CD63 and TSG101, OPN and N‐OPN. HKC‐8 cells were transfected with pDel‐β‐catenin plasmid for 6 h, and then incubated with serum‐free medium for 24 h. Supernatant was collected and used to isolate exosomes. (f) Western blot analyses show N‐OPN protein expression in supernatant and exosome‐removed supernatant in HKC‐8 cells transfected with pDel‐β‐catenin. HKC‐8 cells were transfected with pDel‐β‐catenin plasmid for 6 h, and then incubated with serum‐free medium for 24 h (active β‐cat conditioned medium). Exosomes were removed or not in active β‐cat conditioned medium. (g) Colloidal gold staining demonstrates that both OPN and N‐OPN were encapsulated by exosomes isolated from HKC‐8 cells transfected with pDel‐β‐catenin. Both OPN and N‐OPN were labelled with 10 nm colloidal gold. Arrows indicate OPN and N‐OPN, respectively; scale bar: 100 nm. (h) Experimental design show CD44 was knocked down in NRK‐49F fibroblasts prior to stimulation with tubule‐derived exosomes. HKC‐8 cells were transfected with pDel‐β‐catenin plasmid or pcDNA3 for 6 h, and then incubated with serum‐free medium for 24 h. Supernatant was collected and used to isolate exosomes (active β‐catenin‐Exo or pcDNA3‐Exo). NRK‐49F cells were transfected with siRNA to CD44 for 6 h, and then treated with active β‐catenin‐Exo or pcDNA3‐Exo (30 μg/ml) for 24 h. (i) Western blot analyses show that knockdown of CD44 in NRK‐49F fibroblast cells abolished the expression of fibronectin, α‐SMA, collagen I, active β‐catenin, CD44 and PCNA in NRK‐49F cells after incubation with tubular cell‐derived exosomes (active β‐catenin‐Exo). (j) Double fluorescent staining confirms the intracellular transfer of tubule‐derived exosomal N‐OPN and its colocalization with the receptor of CD44 in NRK‐49F cells. HKC‐8 cells were transfected with pDel‐β‐catenin plasmid for 6 h, and then incubated with serum‐free medium for 24 h. Supernatants were used to isolate exosomes, and incubated with or without Dil (red). HKC‐8 cell‐derived exosomes (30 μg/ml) were incubated into NRK‐49F cells for 12 h, followed by immunofluorescence staining for N‐OPN (green) or CD44 (red) in NRK‐49F cells. Arrows indicate HKC‐8 cell‐derived exosomes and positive staining. Scale bar, 25 μm. (k) Schematic diagram showing the binding site of Foxo4 with OPN. (l, m) Western blot analyses showing the upregulation of Foxo4 protein in HKC‐8 cells transfected with pDel‐β‐catenin. Representative western blot (l) and quantitative data (m) are shown. Numbers (1 to 4) indicate individual treatment in a given group. HCK‐8 cells were transfected with pDel‐β‐catenin plasmid or pcDNA3 for 24 h. **p < 0.01 versus pcDNA3 (*n* = 4). (n) Representative chromatin immunoprecipitation (chip) assay results showing the binding of Foxo4 to the OPN gene promoter region. HKC‐8 cells were transfected with pDel‐β‐catenin or pcDNA3 for 24 h. Cell lysates were precipitated with an antibody against Foxo4, histone H3, or nonimmune IgG, and the ChIP assay was performed for OPN gene promoters. Total diluted lysate was used as the total genomic input DNA. (O) Representative luciferase assay results showing that β‐catenin augmented OPN targeted gene transcription in a Foxo4‐dependent manner. HKC‐8 cells were cotransfected with renilla, pGL3‐OPN, pDel‐β‐catenin, Foxo4 siRNA or OPN siRNA, as indicated. ***p < 0.001 versus pcDNA3 controls, ^###^ *p *< 0.001 versus pDel‐β‐catenin plus Foxo4 siRNA or pDel‐β‐catenin plus OPN siRNA (*n* = 3). (p) Representative western blot showing the expression of active β‐catenin, Foxo4, OPN, N‐OPN and CD44. At 4 days after surgery, UIRI mice were injected with empty vector (pcDNA3) or Wnt1 expression vector (pHA‐Wnt1) through hydrodynamic‐based gene delivery. (q) Colocalization of β‐catenin and OPN are shown using double immunofluorescence staining in Wnt1 overexpressed mice after UIRI. Arrow indicates positive staining; scale bar: 50 μm

To testify the role of β‐catenin in controlling the OPN/CD44 axis, we then transfected HKC‐8 cells with pDel‐β‐catenin plasmid. As shown in Figure 6a and Figure S5D, ectopic expression of β‐catenin induced significant activation of β‐catenin. Furthermore, the expression of CD63, a marker of exosomes, associating with the membranes of intracellular vesicles, was also increased. Interestingly, both OPN and N‐OPN were upregulated, which was concomitant with CD63 induction. However, ectopic expression of β‐catenin did not induce the expression of CD44 (Figure S6P and Q), the receptor of OPN, suggesting that tubular cell derived‐OPN could act on other cells such as fibroblasts, as we have observed the increase in CD44 in interstitial fibroblasts in CKD mice (Figure 2g). We further observed that OPN and N‐OPN were co‐localized with CD63 (Figure 6b). Together, these observations suggest that the generation of extracellular vesicles is accompanied by the induction of OPN and N‐OPN ligands. To further identify the role of β‐catenin in regulating exosomal OPN in tubular cells, we first transfected HKC‐8 cells with siRNA to OPN, and collect the active‐β‐catenin‐CM to treat NRK‐49F cells (Figure 6c). As shown in Figure 6d and Figure S5E–I and M, OPN interference could significantly inhibit active‐β‐catenin‐CM‐induced fibroblast proliferation and activation. To further confirm that OPN was encapsulated by exosomes, we examined exosomes by western blotting and found that active β‐catenin‐Exo contained OPN and N‐OPN. Interestingly, we found that N‐OPN is rarely detected in the supernatant from HKC‐8 cells transfected with pDel‐β‐catenin after removing exosomes, which means that N‐OPN is primarily expressed in exosomes (Figure 6f and Figure S5J). Similarly, colloidal gold staining also showed that OPN and N‐OPN were encapsulated in exosomes, and quite a number of them expressed on the outside membrane of exosomes.

To further investigate the role of tubular cell‐derived exosomal OPN in eliciting its action in recipient cells (NRK‐49F), we transfected NRK‐49F cells with CD44 siRNA or Ctrl siRNA, followed by incubation with pcDNA3‐Exo or active β‐catenin‐Exo (Figure 6h). Active β‐catenin‐Exo induced the upregulation of fibronectin, α‐SMA, collagen I, active β‐catenin, CD44 and PCNA, but these effects were abolished by the knockdown of CD44 (Figure 6i and Figure S6B–G). Then, we labelled exosomes derived from β‐catenin overexpressed‐tubular cells with Dil, a fluorescent lipophilic membrane dye for a long‐term tracing (Liu et al., 2020). Dil‐labelled tubular cell‐derived exosomes were then incubated into NRK‐49F cells. As shown in Figure 6j, Dil‐labelled exosomes were up‐taken by fibroblasts, and co‐localized with N‐OPN. We also found the co‐localization of N‐OPN and its receptor CD44 in NRK‐49F cells (Figure 6j). These further suggests that N‐OPN was encapsulated in tubular cell‐derived esoxomes and transferred to fibroblasts, leading to the activation of fibroblasts via CD44.

Then, we analysed the promoter sequence and found that there was a binding site for Foxo4 in the OPN gene promoter region. As shown in Figure 6k, there were two binding sites for Foxo4 in the OPN gene promoter region. Hence, we assessed HKC‐8 cells transfected with pDel‐β‐catenin and found that Foxo4 expression was increased (Figure 6l and m). Moreover, as shown in Figure 6n, CHIp assay showed that β‐catenin promoted the binding of Foxo4 to the OPN promoter sequence. Consistently, luciferase activity assay showed that β‐catenin induced an increase in OPN transcriptional activity, but treatment with Foxo4 siRNA could significantly reduce this effect (Figure 6o), suggesting that β‐catenin could initiate OPN expression by binding to Foxo4.

Finally, to further confirm the role of β‐catenin in controlling the OPN/CD44 axis in vivo, we injected the Wnt1‐expressing plasmid into UIRI mice. Mouse models of UIRI were described previously (Zhou et al., 2021). The archival kidney samples from earlier studies were used for analyzing. As shown in Figure 6p and Figure S6H–M, ectopic expression of Wnt1 further triggered a significant upregulation of active β‐catenin, Foxo4, OPN, N‐OPN, CD44 and CD63 in UIRI mice, suggesting that β‐catenin enhanced the exosomal OPN/CD44 signal axis in vivo. We further observed that β‐catenin co‐localized with OPN (Figure 6q). Furthermore, in cultured renal tubular cells, ICG‐001, a small molecular compound which inhibits β‐catenin activation, repressed the expression of OPN and N‐OPN in TGF‐β‐treated HKC‐8 cells (Figure S6N and O). These results further revealed that β‐catenin is a master controller for the exosomal OPN/CD44 signal axis.

### 2.6 Tubule‐specific ablation of β‐catenin attenuates renal fibrosis through blocking exosomal OPN/CD44 axis signalling

**FIGURE 7:**
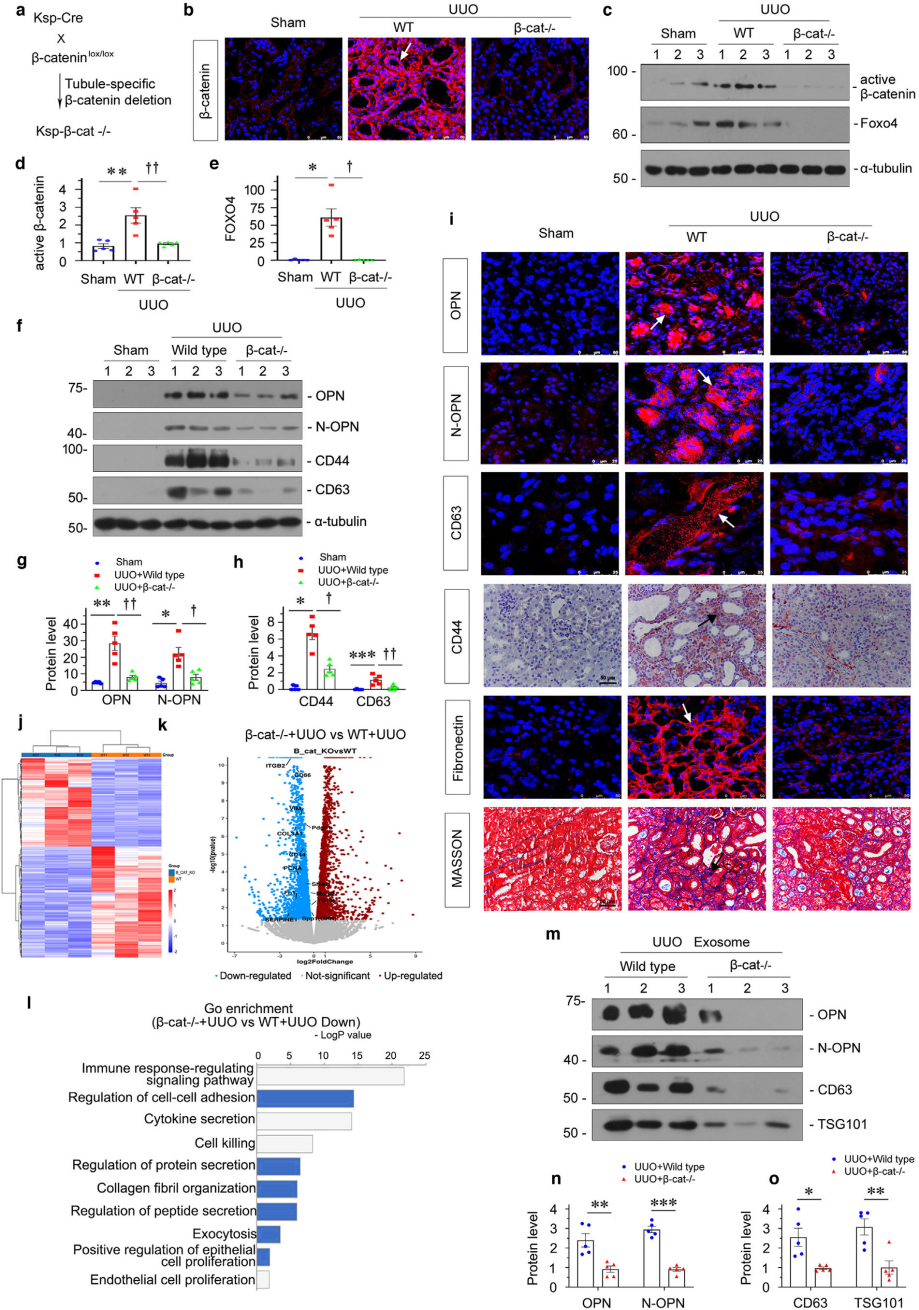
Tubule‐specific ablation of β‐catenin attenuates renal fibrosis through blocking exosomal OPN/CD44 axis signalling. (a) Experimental design showing the strategy of crossbreeding of the β‐catenin‐floxed mice (β‐cat^fl/fl^) with Cre transgenic mice under the control of the Ksp‐cadherin promoter (Ksp‐Cre). (b) Representative immunofluorescence staining of β‐catenin in different groups, as indicated. Arrow indicates positive staining; scale bar: 50 μm. (c–e) Western blot analyses showing renal expression of active β‐catenin and Foxo4 in different groups of mice at 7 days after UUO. Representative western blot (c) and quantitative data of β‐catenin (d) and Foxo4 (e) are shown. *p < 0.05, **p < 0.01 versus sham; ^†^ *p *< 0.05, ^††^ *p *< 0.01 versus WT UUO mice (*n* = 5). (f–h) Western blot analyses showing renal expression of OPN, N‐OPN, CD44 and CD63 in different groups, as indicated. Representative western blot (f) and quantitative data of OPN and N‐OPN (g), and CD44 and CD63 (h) are presented. *p < 0.05, **p < 0.01, ***p < 0.001 versus sham controls; ^†^ *p *< 0.05, ^††^ *p *< 0.01 versus WT UUO mice (*n* = 5). (i) Representative micrographs showing immunofluorescence staining of OPN, N‐OPN, CD63 and fibronectin; immunohistochemical staining of CD44 and collagen deposition assessed by Masson's trichrome staining in different groups, as indicated. Arrows indicate positive staining; scale bar: 50 μm. (j) Gene expression profiling by RNA‐seq shows differential gene clustering of kidney from wild type (WT) UUO mice or tubule specific β‐catenin knockout (β‐cat^−/−^) UUO mice. (k) Volcano plot showing the differentially expressed genes of the two groups, as indicated. (l) Gene ontology (GO) enrichment analysis reveals that several biological processes were enriched, as indicated. (m–o) Western blot analyses showing that the expression of OPN, N‐OPN, CD63 and TSG101 protein in exosomes isolated from WT UUO or β‐cat^−/−^ UUO mice. Exosomes were isolated form kidney tissue of the same weight. Representative western blot (m) and quantitative data of OPN and N‐OPN (n), and CD63 and TSG101 (o) are presented. Numbers (1 to 3) indicates individual animal in a given group. **p < 0.01, ***p < 0.001 versus wild‐type UUO mice (*n* = 5)

We sought to determine the potential role of β‐catenin in regulating OPN/CD44 axis signalling in renal fibrosis. To this end, we generated conditional knockout mice in which the β‐catenin gene is specifically disrupted in renal tubules using the Cre‐LoxP system, according to the previously reported procedures (Figure 7a) (Zhou et al., 2012). We first examined the expression β‐catenin in the kidneys after UUO. As shown in Figure7B, immunofluorescence staining for β‐catenin showed a tubule‐specific reduction of β‐catenin protein in the kidneys of Ksp‐β‐cat^−/−^ mice. Western blot analysis of whole kidney lysate also showed that renal β‐catenin protein was significantly reduced in Ksp‐β‐cat^−/−^ mice compared with wild type (WT) controls (Figure 7c and d). Interestingly, following binding of β‐catenin, Foxo4 protein initiated OPN expression, which decreased significantly after β‐catenin deletion (Figure 7c and e). Therefore, we further explored whether conditional ablation of β‐catenin reduced the expression of OPN. As shown in Figure 7f, g and i, OPN and N‐OPN protein expression levels were markedly decreased in the kidneys of Ksp‐β‐cat^−/−^ mice after UUO compared to controls. Similarly, downregulation of CD44 was also observed in Ksp‐β‐cat^−/−^ mice (Figure 7f, h and i). CD63, a marker of exosomes, was also examined by western blotting in different groups, as indicated. Consistently, CD63 was upregulated in UUO, but decreased following knockout of β‐catenin (Figure 7f, h and i). Furthermore, the loss of β‐catenin also reduced kidney fibrosis (Figure 7i), which is consistent with the previous finding that β‐catenin was harmful in kidney fibrosis.

RNA‐sequencing (RNA‐seq) transcriptome‐clustering profiling of mRNA showed substantial differences between UUO‐affected Ksp‐β‐catenin knockout mice and wild‐type mice (Figure 7j). As shown in Figure 7k, the differential gene expression analysis (volcano plots) showed that OPN, CD44 and other fibrogenesis‐related genes, such as PDGFRB, FN1, PAI‐1, vimentin and collagen IIIa1, were significantly downregulated in β‐catenin knockout mice. Furthermore, GO enrichment analysis showed that the downregulated genes under β‐catenin knockout conditions were assembled in the immune response, cell‐cell adhesion, cytokine secretion, collagen fibril organization, protein secretion, exocytosis and other fibrogene‐correlated pathways (Figure 7l). These results further demonstrated that β‐catenin controlled OPN expression and secretion to further lead to renal fibrosis.

For further confirmation, we then isolated exosomes from the kidneys after UUO and assessed them by western blotting. As shown in Figure 7m–o, the isolated UUO‐affected exosomes contained OPN, N‐OPN, CD63 and TSG101, while they showed a significant reduction after β‐catenin deletion, suggesting that β‐catenin is an effective controller of exosomal OPN/CD44 axis signalling.

### 2.7 Knockout of CD44 decreases kidney fibrosis in the UIRI model

**FIGURE 8:**
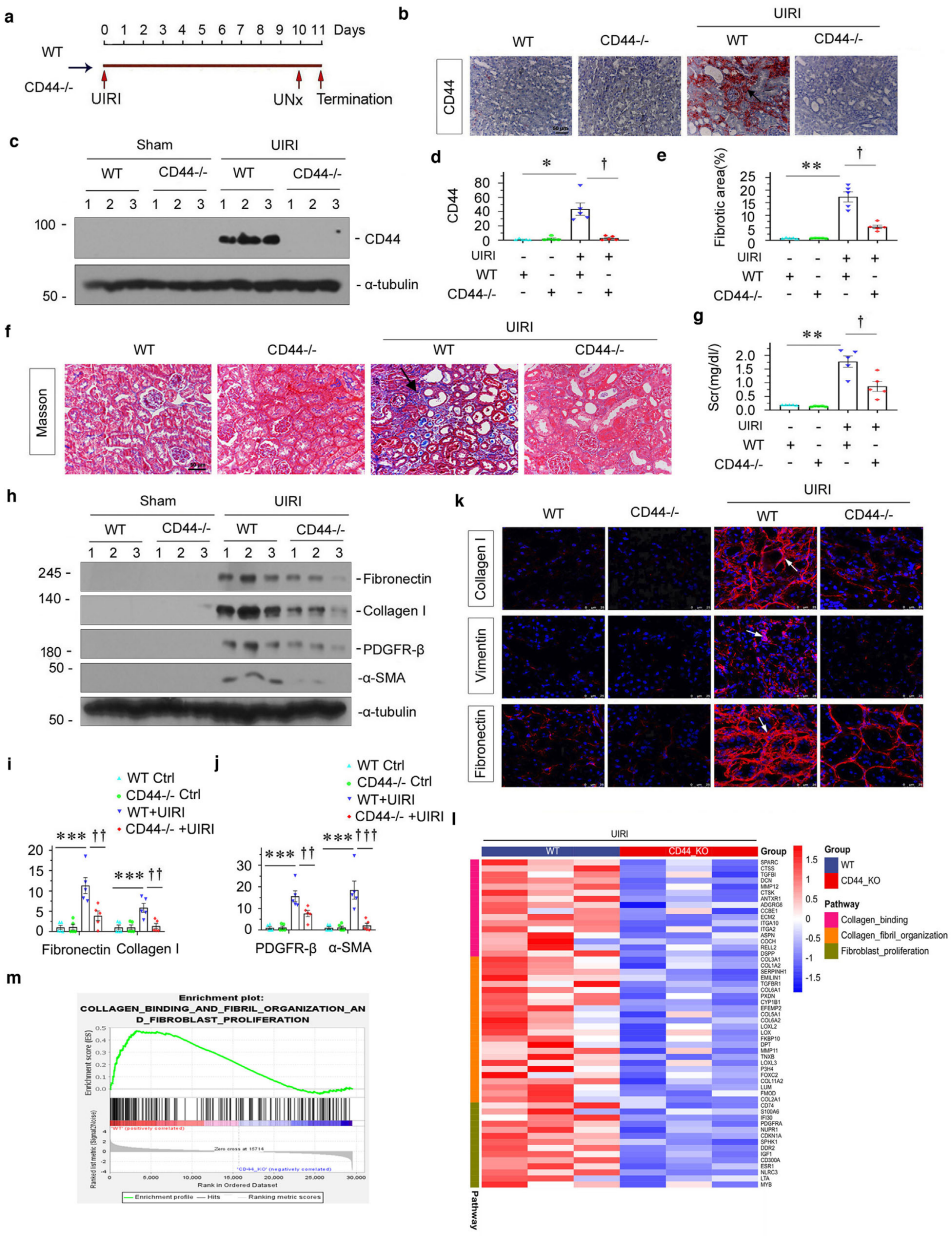
Knockout of CD44 decreases kidney fibrosis in UIRI model. (a) Diagram showing the experimental design. Red arrows indicate the time points when wild‐type mice or CD44 knockout mice undergoing UIRI, UNx and sacrifice. (b) Representative immunohistochemical staining of CD44 in different groups, as indicated. Arrow indicates positive staining; scale bar: 50 μm. (c and d) Western blot shows that knockout of CD44 in mice abolished CD44 expression at 11 days after UIRI. Representative western blot (c) and quantitative data (d) are shown. Numbers (1 to 3) indicate individual animal in a given group. *p < 0.05 versus wild‐type controls; ^†^ *p *< 0.05 versus wild‐type UIRI mice (*n* = 5). (e) Graphic presentation showing the quantitative determination of kidney fibrotic lesions in different groups. **p < 0.01 versus wild‐type controls; ^†^ *p *< 0.05 versus wild‐type UIRI mice (*n* = 5). (f) Representative micrographs show collagen deposition assessed by Masson's trichrome staining in different groups. Arrow indicates positive staining; scale bar: 50 μm. (g) Graphic presentation showing the serum creatinine levels in different groups. **p < 0.01 versus wild‐type controls; ^†^ *p *< 0.05 versus wild‐type mice with UIRI surgery (*n* = 5). (h–j) Western blot showing that knockout of CD44 abolished renal expression of fibrosis‐related proteins in mice at 11 days after UIRI. Representative western blot (h) and quantitative data on fibronectin and collagen I (i), and PDGFR‐β and α‐SMA (j) are shown. Numbers (1 to 3) indicate individual animal in a given group. ***p < 0.001 versus wild‐type controls; ^††^ *p* < 0.01, ^†††^ *p* < 0.001 versus wild‐type mice with UIRI surgery (*n* = 5). (k) Representative micrographs show the expression of collagen I, vimentin and fibronectin in different groups, as indicated. Kidney sections were stained with antibodies against collagen I (upper), vimentin (middle) and fibronectin (bottom), respectively. Arrows indicate positive staining; scale bar: 50 μm. (l) Gene expression profiling by RNA‐seq shows differential gene clustering of kidney from wild type (WT) UIRI mice or CD44 (CD44^−/−^) knockout UIRI mice. (m) GSEA analysis shows the collagen binding and fibril organization and fibroblast proliferation decreased in CD44^−/−^ UIRI mice compared with WT UIRI mice

To discern the role of CD44 in renal fibrosis, we constructed global CD44 knockout mice (CD44^−/−^). The experimental design is presented in Figure 8a. The efficacy of CD44 knockout was confirmed by both immunohistochemistry (Figure 8b) and western blotting (Figure 8c–d). To test the role of CD44 in renal fibrosis, we counted renal interstitial fibrotic lesions after histological Masson staining. As shown in Figure 8e and f, interstitial fibrotic lesions, manifested by the deposition of collagen and fibronectin, were obviously aggravated after UIRI injury, but were further blocked by knockout of CD44. Next, Scr was assessed to further confirm the role of CD44 in CKD. As shown in Figure 8g, the Scr level was significantly elevated after UIRI. However, knockout of CD44 significantly attenuated the Scr increase in UIRI mice. Furthermore, we assessed fibrogenesis‐related protein expression by western blotting and immunofluorescence. As shown in Figure 8h–k, fibronectin, collagen I, PDGFR‐β, α‐SMA and vimentin expression levels were induced in UIRI mice, but they were apparently inhibited under the CD44 knockout condition in this model. These data suggest that a lack of CD44 retarded kidney fibrosis in CKD.

To further identify the role of CD44 in renal fibrosis, we performed RNA sequencing in UIRI‐affected wild‐type mice and CD44 knockout mice. As shown in Figure 8l, knockdown of CD44 alleviated collagen binding, fibril organization and fibroblast proliferation, the three important processes of organ fibrosis. GSEA also demonstrated the detrimental role of CD44 in these important fibrogenesis‐related pathways (Figure 8 m).

### 2.8 Tubular cell‐derived exosomal OPN plays a central role in promoting renal fibrosis

**FIGURE 9:**
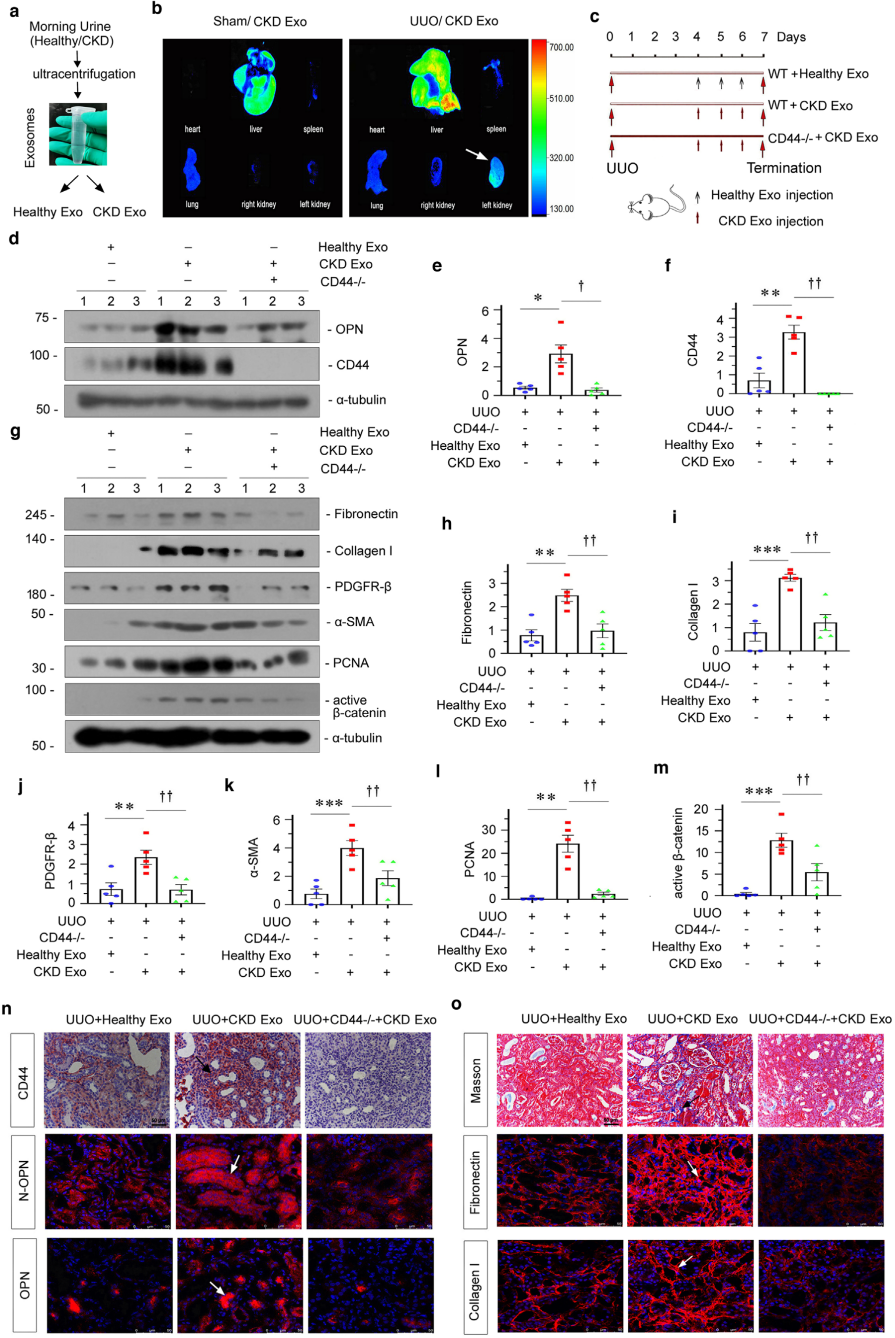
Tubular cell‐derived exosomal OPN plays a central role in promoting renal fibrosis. (a) Schematic diagram showing obtaining exosomes. (b) Urinary exosomes from patients with CKD were labelled with fluorescent lipophilic membrane dye Dil. Mice were injected by tail vein with urinary exosome from patients with CKD. Fluorescent images of organs were acquired using a Bruker Small Animal Optical Imaging System. Sham (left) and CKD mice (right) were injected with exosome labelled with Dil by the tail vein. The fluorescence luminescence of the heart, lung, liver, spleen and kidney was assessed at 12 h after injection. (c) Experimental design. Large red arrows indicate the time points undergoing UUO and termination, respectively. Small black arrows indicate the time points when exosomes derived from the urine of healthy subjects (Healthy‐Exo 200 μg) were injected intravenously. Small red arrows indicate when exosomes derived from urine of CKD patients (CKD‐Exo 200 μg) were injected intravenously. (d–f) Western blot showing that knockout of CD44 in mice injected with exosomes derived from patients with CKD abolished OPN and CD44 expression at 7 days after UUO. Representative western blot (d) and quantitative data of OPN (e) and CD44 (f) are shown. Numbers (1 to 3) indicate individual animal in a given group. *p < 0.05, **p < 0.01 versus UUO mice treated with Healthy‐Exo; ^†^ *p* < 0.05, ^††^ *p* < 0.01 versus UUO mice treated with CKD‐Exo (*n* = 5). (g–m) Western blot analyses showing renal expression of fibronectin, collagen I, PDGFR‐β, α‐SMA, PCNA and active β‐catenin in different groups, as indicated. Representative western blot (g) and quantitative data of fibronectin (h), collagen I (i), PDGFR‐β (j) and α‐SMA (k), PCNA (l) and active β‐catenin (m) are shown. Numbers (1 to 3) indicate individual animal in a given group. **p < 0.01, ***p < 0.001 versus UUO mice treated with Healthy‐Exo; ^††^ *p* < 0.01 versus UUO mice treated with CKD‐Exo (*n* = 5). (n) Kidney tissues from different groups were subjected to immunostaining for CD44 (upper), immunofluorescence staining of N‐OPN (middle) and OPN (bottom). Images demonstrate that knockout of CD44 in UUO mice injected with exosomes from patients with CKD abolished OPN and N‐OPN expression in mice at 7 days after UUO. Arrow indicates positive staining; scale bar: 50 μm. (o) Representative micrographs showing collagen deposition, as well as fibronectin and collagen I expression in different groups, as indicated. Kidney sections were subjected to Masson's trichrome staining for collagen deposition (upper), and immunofluorescence staining of fibronectin (middle) and collagen I (bottom). Arrow indicates positive staining; scale bar: 50 μm

To investigate the role of tubule‐derived exosomes in renal fibrosis, we conducted animal studies using the UUO model by injection of the urinary exosomes isolated from patients with CKD (CKD‐Exo) or healthy controls (Healthy‐Exo), as shown in Figure 9a. We first testified whether exosomes located to diseased kidney after tail vein injection (Liu et al., 2020; Tang et al., 2020). As shown in Figure 9b, in UUO mice, fluorescence could be observed not only in liver, spleen and lung, but also highly in left kidney suffering from ureteral ligation; while it was weakly observed in right kidney. However, in sham mice, fluorescence was observed in the majority in liver and lung. This suggests that UUO‐affected kidney had adhesive ability to CKD‐Exo, further implying the interaction between CKD‐Exo components with local cells. To testify the underlying mechanisms, we injected CKD‐Exo to UUO‐affected CD44 knockout mice. The experimental design is shown in Figure 9c. On days 4, 5 and 6, UUO mice were given the same amount of Healthy‐Exo or CKD‐Exo. As shown in Figure 9d–f, injection of CKD‐Exo induced OPN and CD44 protein expression, which was inhibited by genetic ablation of CD44. Similarly, immunohistochemical and immunofluorescence staining also revealed a substantial increase in CD44, OPN and N‐OPN protein in the obstructed kidneys 7 days after UUO, but this effect was largely blocked in the absence of CD44 (Figure 9n).

**FIGURE 10:**
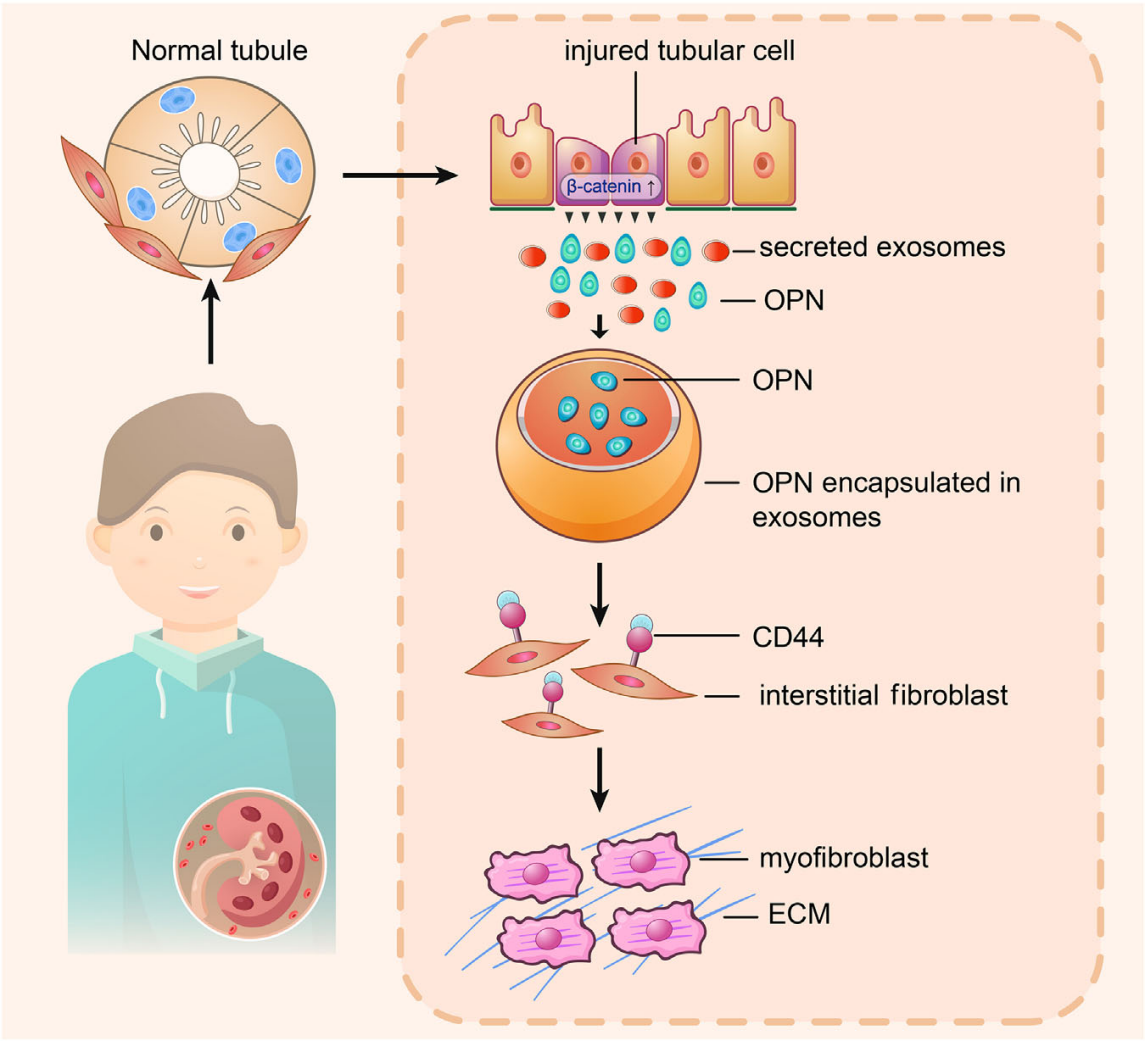
Working model. The schematic presentation depicts the potential mechanism by which exosomal OPN/CD44 axis meditates renal fibrosis. β‐catenin signal is induced in injured tubular cells, and then promotes secretion of exosomes and OPN expression. Tubular cell‐derived OPN is encapsulated in exosomes, and then transferred to bind with the receptor of CD44 in interstitial fibroblasts. The communication between tubular cells and fibroblasts via exosomal OPN/CD44 axis activates myofibroblast activation and promotes the development and progression of renal fibrosis

We then examined the effects of these treatments on renal fibrotic lesions. As shown in Figure 9g–m, fibronectin, collagen I, PDGFR‐β, α‐SMA, PCNA and active β‐catenin expressional levels were significantly upregulated in UUO mice; however, knockout of CD44 alleviated the expression of these proteins. Consistently, Masson and immunofluorescence staining showed that CKD‐Exo injection aggravated fibronectin and collagen deposition in UUO mice, compared with Healthy‐Exo. However, knockout of CD44 largely abolished the effects of CKD‐Exo (Figure 9o). These data suggest that tubular cell‐derived exosomal OPN plays a central role in promoting renal fibrosis through CD44 signalling.

Collectively (Figure 10), our results suggest that β‐catenin is a master controller for tubular cell and interstitial fibroblast communication, which promotes exosome secretion and OPN expression in injured tubular cells. OPN is encapsulated in tubular cell‐derived exosomes, and then transferred to interstitial fibroblasts and induces myofibroblast activation through binding to its receptor CD44. Exosome‐mediated activation of OPN/CD44 axis leads to the development and progression of renal fibrosis. Of note, β‐catenin is the key causative factor for these series of processes.

## 3 DISCUSSION

CKD is becoming a public health problem, which affects more than 10% of the global population (Huang et al., 2021). Although initiated by various aetiologies, renal fibrosis is a final common pathological change in all types of CKD (Gewin, 2018; Jing et al., 2020). Although therapies such as dialysis and transplantation can ameliorate or delay the progression of renal fibrosis, there are currently no curative strategies (Nastase et al., 2018). The main reason for the lack of curative treatment is the complicated mechanisms involved in CKD and renal fibrosis, which have not been fully elucidated yet.

Tubules and interstitium are important components in kidneys. Renal tubular cells execute functions of reabsorption, secretion and excretion. With the high‐energy requirement and active metabolism, tubular epithelial cells are believed to be the primary targets and initial reactors after kidney injury. The maladaptive repair of the tubular epithelium is considered to be a key step of kidney fibrosis (Qi & Yang, 2018). Residing in the interstitium and connecting the adjacent tubules, renal interstitial fibroblasts provide important structural support and facilitate tissue remodelling through modulation of extracellular matrix components. As a result, they are considered as the final executor in the development of kidney fibrosis (Qi & Yang, 2018). Furthermore, as the neighbouring cells, tubular epithelial cells and fibroblasts commonly crosstalk and transmit information to aid kidney repair in physiological condition or aggravate disease progression under maladaptive conditions (Qi & Yang, 2018). Notably, defective communication plays a central role in the activation of kidney fibrosis (Nastase et al., 2018). Besides soluble factors (Qi & Yang, 2018), numerous signalling proteins, RNAs and lipids are encapsulated into extracellular vesicles such as exosomes, to be more efficiently transported, and to a greater extent, to prevent their rapid degradation (Cao et al., 2021; He et al., 2018; Wortzel et al., 2019). Interestingly, recent studies have suggested that the communication between tubular cells and interstitial fibroblasts, especially through exosomal transmission, plays a crucial role in mediating tubular cell EMT and fibroblast activation in renal fibrogenesis, as reported by our group and others (Jing et al., 2019; Liu et al., 2020).

Exosomes are released by most types of cells, and they are surrounded by a double‐layer membrane from the original cells by exocytosis (Liu et al., 2020). Through transmitting a variety of molecules, exosomes play an important role in intercellular communication to modulate the behaviour of recipient cells by receptor‐ligand interactions, direct membrane fusion or endocytosis (He et al., 2018; Pegtel & Gould, 2019; Tang et al., 2020). There has been a lot progress in understanding the role of exosomes in renal diseases in recent years (Cao et al., 2021; Feng et al., 2021; Li et al., 2019; Lv et al., 2020; Lv et al., 2018; Tang et al., 2020), however, the internal signal transmission mechanisms still need to be studied in detail.

In the present study, we found that tubular cell‐derived exosomes induced fibroblast activation in vitro, which was abolished through blockade of exosome biogenesis and secretion by DMA (Figure 5 and Figure S4). Similarly, kidney fibrosis was also inhibited by the prevention of exosome secretion in vivo (Figures 3 and 4), suggesting that exosomes serve as unique and powerful carriers for signal exchange between tubular cells and fibroblasts, and to further promote the development and progression of renal fibrosis.

A novel discovery of this study is that OPN and N‐OPN were enriched in urinary exosomes isolated from CKD patients (Figure 1), diseased kidneys models (Figure 2) and tubular cells with overexpression of β‐catenin (Figure 6), a detrimental mediator to renal fibrosis. These results suggest that OPN and its terminal fragment, N‐OPN, play an important role in the progression of renal fibrosis through the secretion of exosome. Although some reports have suggested that OPN is involved in ECM accumulation and contributes to kidney fibrosis (Vianello et al., 2020; Yoo et al., 2006), how OPN is transported to the recipient cell remains a mystery. We observed that OPN was primarily expressed in proximal and distal renal tubular epithelial cells, while its receptor CD44, was mainly located in interstitial fibroblasts (Figure 2). CD44 is a cell surface receptor that regulates cell proliferation, adhesion, migration and invasion, and could bind to several ligands, including hyaluronic acid and OPN (Chen et al., 2018; Krolikoski et al., 2019; Xiao et al., 2011). The binding of OPN and CD44 plays roles in bone remodelling, stem cell migration and neuron development (Chellaiah et al., 2003; Lee et al., 2020; Plantman, 2012). However, their involvement in renal fibrosis has been scarcely reported.

In this study, we found that exosomes, the powerful carriers for biomolecules, transported renal tubular epithelial cell‐derived OPN and N‐OPN to the receptor CD44 in interstitial fibroblasts. Several lines of evidences supported this finding. First, knockdown of CD44 by siRNA in NRK‐49F cells abolished cell activation induced by renal tubular cell‐derived exosomal OPN (Figure 6 and Figure S6). Furthermore, exosomes isolated from the urine of patients with CKD promoted the activation of fibroblasts in UUO mice, but this was blocked in UUO mice with CD44 gene knockout (Figure 9).

Another novel and interesting finding of this study is that N‐OPN serves as an indicative marker for CKD progression and renal fibrosis. Because invasive kidney biopsy is still the only criterion for assessing renal fibrotic damage, it is necessary to develop a noninvasive biomarker as an alternative to assess, predict and monitor the progression of renal fibrosis in patients with CKD. Notably, we found that urinary N‐OPN serves as a biomarker in renal fibrogenesis. Moreover, urinary OPN was already significantly increased in patients with normal renal function (eGFR > 90 ml/min per 1.73 m^2^, CKD 1 stage) (Figure 1). This suggests that urinary N‐OPN is an early noninvasive biomarker that indicates the ongoing fibrogenesis. In patients with CKD stages 2–5, urinary N‐OPN did not gradually increase compared to that in patients with stage 1 CKD (Figure 1j). This may be due to the severe damage of renal tubular epithelial cells following the progression of CKD, at which point, no further N‐OPN can be secreted.

β‐catenin, an effector of the canonical Wnt signalling, plays a crucial role in kidney development and fibrotic diseases (Conduit et al., 2019). In our previous studies, we found that the reactivated β‐catenin pathway is intimately related to tubular cell senescence and fibroblast activation, both of which are important contributors to renal fibrosis (Miao et al., 2019; Xiao et al., 2016; Zhou et al., 2013). Although previous studies have shown that OPN promotes progenitor cell expansion and tumorigenicity via activation of β‐catenin (Lin et al., 2020), and that β‐catenin could be a transcriptional coactivator for OPN expression in cancer (Yang et al., 2017), the relationship between β‐catenin and OPN in renal fibrosis is unclear. In our study, we found that exosomal OPN is controlled by β‐catenin in kidney diseases. There are several lines to support this view. Firstly, the results of both ChIP assay and luciferase activity assay showed that β‐catenin promoted the binding of the OPN promoter sequence with Foxo4, another important transcription factor for renal fibrosis (Figure 6). In addition, tubular cell‐specific ablation of β‐catenin reduced the expression of OPN both in situ in kidney tissue and the isolated exosomes (Figure 7). Finally, overexpression of β‐catenin in tubular cells enhanced the secretion of exosomes containing OPN (Figure 6). These findings, for the first time, underscore a pivotal role of β‐catenin in regulating exosomal OPN in the field of kidney disease.

We also found that CD44 induced the upregulation of multiple Wnt(s) in fibroblasts. Furthermore, the conditioned medium from CD44‐overexpressed fibroblasts greatly triggered the activation of β‐catenin, and the upregulation of OPN and N‐OPN. While in fibroblasts, CD44 could only promote a weak expression of OPN (Figure S7). These further suggest that OPN/CD44 axis mediates the communication between tubular cells and fibroblasts through coordinating with Wnt/β‐catenin signalling. Hence, this explains why OPN could also be inhibited in CD44 KO mice (Figure 9), suggesting the reciprocal loop between tubular cells and fibroblasts, that is, β‐catenin triggers the exosome secretion from tubular cells and OPN expression in exosomes, which activates CD44 in fibroblasts and further triggers Wnt release to promote β‐catenin activation in tubular cells. More studies should be performed to verify the theory, however, this investigation is beyond the scope of the current study.

In summary, our results showed that exosomes play an important role in promoting renal fibrosis by mediating communication between tubular epithelial cells and fibroblasts. Moreover, a large number of exosomes are induced in tubular epithelial cells after kidney injury, with contents including OPN and N‐OPN. Finally, exosome‐mediated OPN/CD44 axis activation is controlled by β‐catenin signalling. This study provides a novel mechanistic linkage of β‐catenin signalling and exosomal OPN/CD44 axis in the setting of renal fibrosis, and underscores an important therapeutic strategy for CKD.

## 4 METHODS AND MATERIALS

### 4.1 Human kidney biopsies and urine samples

All human samples (urine and kidney biopsies) were collected from patients with CKD at Ruikang Hospital following provision of written consent. The demographic and clinical data are presented in Table S1. Human specimens were obtained from kidney biopsies with clinic diagnostic. Per 50 ml of the clean morning urine samples were collected from 183 patients with CKD with diagnostic biopsy or clinically diagnosed and 30 healthy volunteers. All of the studies involving human samples were approved by the Ethic Committee on Human Subjects of Ruikang Hospital, Guangxi University of Traditional Chinese Medicine (KY2019‐005).

### 4.2 OPN N‐half enzyme‐linked immunosorbent assay

Human OPN N‐half Assay Kit was purchased from the ImmunoBiological Laboratories (IBL Company, Cat. JP27258). Human urinary OPN N‐half levels were measured according to the procedures outlined by the manufacturer.

### 4.3 4D‐Label free proteomics analysis

The primary experimental procedures for 4D‐Label free proteomics analysis include protein extraction, trypsin digestion, LC‐MS/MS analysis and data analysis. The resulting MS/MS data were processed using the MaxQuant search engine (v.1.6.15.0). Tandem mass spectra were searched against the Rat UniProt database (29,940 entries) concatenated with a reverse decoy database. GO enrichment analysis of the differentially expressed protein in exosomes from patients with CKD and OPN‐interacted proteins was identified by the clusterProfiler R package. The OPN‐interacted protein was analysed by STRING database and mapped using Cytoscape software. The proteomics analysis in our research is supported by Jingjie PTM BioLabs (Hangzhou, China).

### 4.4 Animal models

Male C57BL/6 mice were purchased from the Experimental Animal Centre of Southern Medical University (Guangzhou, China). Tubule‐specific β‐catenin conditional knockout mice (Ksp‐β‐catenin^−/−^) were created by mating β‐catenin floxed mice with Ksp‐cre transgenic mice, which were purchased from Cyagen Biosciences (stock no. CKOCMP‐12387‐Ctnnb1‐B6N‐VA; Cyagen Biosciences, Guangzhou, China). The CD44 null mice (CD44^−/−^) aged 6–8 weeks, on a C57BL/6N background, were also purchased from Cyagen Biosciences (stock no. KOCMP‐12505‐Cd44‐B6N‐VA). The genotyping of tail DNA samples was performed using a routine PCR protocol as previously reported (Zhou et al., 2012). For the UUO model, after a midline abdominal incision, the left ureter was separated from the surrounding tissues and mice received double ligation of the left ureter. For the UIRI model, the left renal pedicle was clipped for 35 min, with the body temperature maintained at 37.8°C. On the 10th day, the right kidney was resected, and the mice were sacrificed at 11 days post‐UIRI. In vivo expression of OPN and inhibition of exosome secretion in mice were performed using a hydrodynamic‐based gene delivery approach and daily intraperitoneal injections of DMA at a dose of 10 mg/kg body weight. The detailed experimental designs are shown in Figures 3a and 4a. For studying the effects of exosomes, CD44^−/−^ mice were used and the experimental design is detailed in Figure 9a. Sterile exosomes were collected from healthy subjects or patients with CKD, quantified using the microbicinchoninic acid (BCA) protein assay, and injected intravenously (200 μg per mouse per time point). The animal experiments were approved by the Animal Ethics Committee of Southern Medical University, Guangzhou, China (NFYY‐2019‐1118).

### 4.5 Serum creatinine and BUN measurement

Serum creatinine and BUN levels were determined by an automatic chemistry analyser (AU480, Beckman Coulter, Pasadena, California). The data are shown as mg/dl.

### 4.6 TEM and colloidal gold electron microscope

The handling and detection of electron microscopic samples were performed by the kidney electron microscopic core laboratory of Guangzhou KingMed Center for Clinical Laboratory Co., Ltd (Guangzhou, China). For TEM, pelleted exosomes or small pieces of kidney tissue were examined according to procedures established at the KingMed Center. For colloidal gold electron microscopy, exosomes were first adsorbed onto electron‐microscope grids, rinsed twice in PBS, and incubated with 2% BSA for 10 min. Then, OPN antibody (Boster Biotechnology, Cat. PB0589, 1:50) or N‐OPN antibody (Abcam, Cat. ab181440, 1:50) was added and incubated overnight at 4°C. Following incubation, the grids were rinsed in PBS six times and incubated with 2% BSA for 10 min. Finally, gold‐IgG diluted in 2% BSA was added to the samples and incubated in a 37°C incubator for 60 min. Following incubation, the samples were rinsed in distilled water six times, stained with 2% uranyl acetate for 5–10 min, and rinsed once with water. We then waited for the grid to dry and prepared labelled exosomes for electron microscopy (JEM‐1400 PLUS, Tokyo, Japan).

### 4.7 Cell culture and treatment

HKC‐8 cells were transfected with pDel‐β‐catenin for 4–6 h, followed by incubating in serum‐free medium. In some experiments, HKC‐8 cells were pretreated with 100 μmol/L DMA or cotransfected with OPN siRNA using Lipofectamine 2000. NRK‐49F cells were treated with conditioned media from HKC‐8 cells or exosomes (pcDNA3‐Exo or active β‐cat‐Exo, 30 μg protein/ml) isolated from HKC‐8 cells transfected with or without pDel‐β‐catenin. In some experiments, NRK‐49F cells were pretreated with human recombinant OPN (Boster Biotechnology, Cat. PROTP10451) at 50 ng/ml or transfected with CD44 siRNA or CD44 expression plasmid using Lipofectamine 2000.

### 4.8 Exosome isolation

Differential centrifugation at 4°C was used to isolate exosomes. The differential centrifugation process was as follows: morning urine was centrifuged at 300 g for 5 min to collect the supernatant, then, 2000 g for 20 min to collect the supernatant, and 10,000 g for 30 min to collect the supernatant, after removing the cell and debris, followed by filtration with a 0.22μm filter to remove microvesicles. For exosome isolation and purification, the supernatant was ultracentrifuged at 110,000 × *g* for 2 h (Type 70 Ti rotor, Beckman Coulter Optima L‐XP) at 4°C. Pellets (exosomes) were washed to eliminate contaminating proteins and centrifuged again at 110,000 × *g*, and resuspended with sterile 1 × PBS buffer, and quantified the numbers.

For exosome isolation from conditioned media, the cells were transfected with indicated plasmids, washed with sterile PBS, and then incubated for an additional 24 h in serum‐free medium. Conditioned media was collected to isolate exosomes using differential centrifugation as abovementioned.

### 4.9 Western blot analysis

Protein expression was analyzed by western blot analysis as described previously (Zhou et al., 2015). The following primary antibodies were used: anti‐N‐OPN (Abcam, Cat. ab181440, 1:1000), anti‐CD63 (Abcam, Cat. ab59479, 1:1000), anti‐OPN (Boster Biotechnology, Cat. PB0589, 1:1000), anti‐CD44 (Boster Biotechnology, Cat. A00052, 1:1000), anti‐α‐tubulin (Beijing Ray Antibody Biotech, Cat. RM2007, 1:5000), anti‐fibronectin (Sigma, Cat. F3648, 1:50000), anti‐α‐SMA (Abcam, Cat. ab5648, 1:1000), anti‐PDGFR‐β (Santa Cruz, Cat. sc‐374573, 1:1000), anti‐Collagen I (Boster Biotechnology, Cat. BA0325, 1:1000), anti‐Vimentin (Abcam, Cat. ab8978, 1:1000), anti‐PCNA (Abcam, Cat. ab29; 1:1000), anti‐active‐β‐catenin (Cell Signaling, Cat. #4270s, 1:1000), anti‐c‐Myc (Cell Signaling, Cat. #5605s, 1:1000), and anti‐Foxo4 (Cell Signaling, Cat. #9472s, 1:1000), anti‐TSG101 (Abcam, Cat. Ab83; 1:1000), anti‐CD81 (Boster Biotechnology, Cat. A01281‐2, 1:1000), anti‐Alix (Boster Biotechnology, Cat. BM5496, 1:1000), anti‐Flag (Boster Biotechnology, Cat. M30971, 1:1000).

### 4.10 Chromatin immunoprecipitation (ChIP)

HKC‐8 cells were transfected with active β‐catenin expression plasmid (pDel‐β‐catenin) for 24 h. Cells were fixed with 4% formaldehyde for 10 min at room temperature for protein‐DNA crosslinking. Cell lysates were obtained and the ChIP assay was performed using the SimpleChIP Plus (Magnetic Bead) Kit (Cell Signaling, Cat. 9005). The antibody against Foxo4 (Cell Signaling, Cat. #9472s), H3, and normal rabbit IgG was added and incubated overnight at 4°C, followed by incubation with protein A‐agarose for 1 h. After washing out the precipitate, purified DNA was used as a template for PCR. The sequences of human OPN primers were as follows: forward 5′‐aaggaagctgacactttagg‐3′ and reverse 5′‐gagctctgggtccttttaaa‐3′.

### 4.11 Transcriptomic analysis

Total RNA was extracted according to the manufacturer's instructions. The primary experimental procedures for transcriptome sequencing analysis include RNA quantification and qualification, library preparation for transcriptome sequencing, clustering and sequencing, and data analysis. HTSeq v0.6.0 was used to count the number of reads mapped to each gene. The FPKM of each gene was calculated based on the length of the gene and the read count mapped to this gene. Differential expression analysis of the two groups was performed using the DESeq2 R package (1.10.1). GO enrichment analysis of differentially expressed genes was performed using the clusterProfiler R package. The GSEA web interface with the Molecular Signatures Database ‘collagen binding’, ‘collagen fibril organization’ and ‘fibroblast proliferation’ genesets was used to reveal the formation process of kidney fibrosis. Adjusted *p *< 0.05 was considered significantly differential expression. The transcriptome sequencing analysis in our research was supported by Novogene Co., Ltd (Beijing, China).

### 4.12 Luciferase assay

The effect of β‐catenin on Foxo4‐mediated gene transcription was assessed using a luciferase reporter kit (E1910, Promega, Madison, WI). Briefly, HKC‐8 cells were seeded in a 6‐well plate and cotransfected using Lipofectamine 2000 reagent with pGL3‐OPN and active β‐catenin expression vector (pDel‐β‐catenin) in the absence or presence of siRNA to Foxo4 or OPN, as indicated. Renilla luciferase was used as an internal control reporter. The luciferase assay was conducted using a dual luciferase assay system kit (Promega, Cat. E1910) according to the manufacturer's protocols. Relative luciferase activity (arbitrary units) was calculated as fold induction over the controls after normalizing the transfection efficiency.

### 4.13 Immunofluorescence staining

Frozen sections of kidneys were fixed with 4% paraformalin‐fixing solution for 15 min at room temperature. NRK‐49F cells cultured on coverslips were fixed with cold methanol:acetone (1:1) for 15 min at room temperature, followed by blocking with 10% normal donkey serum in PBS. Slides were incubated with primary antibodies against anti‐N‐OPN (Abcam, Cat. ab181440, 1:50), anti‐OPN (Boster Biotechnology, Cat. PB0589, 1:50), anti‐CD63 (Abcam, Cat. ab59479, 1:50), anti‐Lotus Tetragonolobus Lectin (LTL) (VECTOR Laboratories, Cat. FL‐1321,1:400), anti‐Peanut Agglutinin (PNA) (VECTOR Laboratories, Cat. FL‐107, 1:400), anti‐Dolichos Biflorus Agglutinin (DBA) (VECTOR Laboratories, Cat. FL1031, 1:400), anti‐Collagen I (Boster Biotechnology, Cat. BA0325, 1:50), anti‐Vimentin (Abcam, Cat. ab8978, 1:50), anti‐fibronectin (Sigma, Cat. F3648; 1:100), anti‐CD44 (Boster Biotechnology, Cat. M00052‐3, 1:50) and anti‐β‐catenin (BD Biosciences, Cat. 610154, 1:150). After washing with TBS‐T, slides were incubated with Cy2 or Cy3‐conjugated donkey anti‐ or anti‐rabbit IgG (Jackson Immuno‐Research Laboratories, West Grove, PA). Nuclei were stained with DAPI (Beyotime, Cat. C1006) according to the manufacturer's instructions. All images were taken by confocal microscopy (Leica TCS SP2 AOBS, Leica Microsystems, Buffalo Grove, IL).

### 4.14 EdU‐based cell proliferation assay

The proliferation of NRK‐49F cells was determined using the Click‐iT plus EdU Alexa Fluor 488 Imaging Kit (Thermo Fisher Scientific). Briefly, cells were treated as indicated and incubated with 10 μM EdU for 6 h before fixation and permeabilisation. Finally, the cells were stained with EdU reaction solution. Cell nuclei were counterstained with Hoechst 33258 (Beyotime, Cat. C0003‐2) for 30 min. Images were taken by confocal microscopy (Leica TCS SP2 AOBS, Leica Microsystems, Buffalo Grove, IL).

### 4.15 Histology and immunohistochemical staining

Paraffin‐embedded (4‐μm thick) kidney sections were prepared using a routine procedure. Sections were stained with Masson staining to identify collagen deposition. Computer‐aided technology was used to quantify fibrotic damage (Zhou et al., 2021). Immunohistochemical staining was performed using a routine protocol. The following antibodies were used: anti‐CD44 (Abcam, Cat. ab189524, 1:80), anti‐N‐OPN (Abcam, Cat. ab181440, 1:50). Images were taken by an Olympus DP80 microscope with EMCCD camera.

### 4.16 Nanoparticle tracking analysis (NTA)

NTA was performed using the ZetaView Particle Metrix (Meerbusch, Germany). Isolated exosomes were diluted using 1 × PBS buffer to measure particle size and concentration. Data from the ZetaView were analysed using the software ZetaView 8.04.02. According to the measured concentration, resuspension volume and dilution factor were used to calculate the absolute numbers of particles.

### 4.17 Organ imaging of exosome distribution

C57BL/6 mice were subjected to Sham or UUO surgery. Five days after surgery, mice were intravenously injected with 400 μl of Dil‐labelled urinary exosomes (200 μg) from CKD patients. All mice were sacrificed after 12 h. Major organs including kidneys, heart, liver, lung and spleen were removed and placed in glass dishes. Organs were exposed to a Bruker FX PRO imaging system equipped with an excitation at 549 nm and emission at 565 nm, and images were taken with camera and analyzed digitally. All procedures were conducted in dark.

### 4.18 Exosome quantification

EXOCET Exosome Quantitation Assay Kit was purchased from the System Biosciences (SBI Company, Cat. EXOCET96A‐1). Human urinary exosome numbers were measured according to the manufacturer's protocols, by measuring the activity of exosome acetylcholinesterase.

### 4.19 Labeling of urinary exosome or exosomes from conditioned media

To obtain Dil (1,1′‐dioctadecyl‐3,3,3′,3′‐tetramethylindocarbocyanine perchlorate)‐labelled exosomes, purified urinary exosome from patients with CKD or conditioned media‐derived exosomes were incubated in the presence of 5 μl/ml Dil fluorescent dye (V22885, Invitrogen) for 30 min at 37 °C, then resuspended in 30 ml of PBS and ultracentrifuged at 200,000 × *g* for 2 h to remove Dil dye. After being washed twice, the labelled urinary exosomes were resuspended in PBS; active‐β‐catenin‐ exosomes were resuspended in serum‐free medium and then add to fibroblasts and incubate cells for 12 h.

### 4.20 Real‐Time qRT‐PCR

Total RNA was isolated using TRIzol RNA isolation system (Life Technologies, Grand Island, NY) according to the manufature instruction. The cDNA synthesis was carried out by using a Promega reverse transcription (Promega, Madison, WI), and qRT‐PCR was performing on an ABI prism 7000 Sequence Detection System (Applied Biosystems, Foster City, CA). The sequences of the primer pairs for different genes are described in the Table S2. The mRNA levels of various genes were calculated after normalizing with β‐actin.

### 4.21 Silver staining

First morning urine was collected from CKD patient. Urine sample (50 ml) was passed through filter paper and centrifuged at 300 × *g* for 5 min at room temperature (RT) to collect sediment and supernatant. Urinary exosomes were collected as described earlier. Protein concentration was determined using a BCA Protein Quantification Kit. Fast Silver Stain Kit was purchased from the Beyotime (Cat. P00175, Shanghai, China). Silver staining was measured according to the procedures outlined by the manufacturer.

### 4.22 Iodixanol density gradient centrifugation

Gradient fractions were prepared by diluting 60% iodixanol (OptiPrep, Serumwerk Bernburg AG for Alere Technologies) with PBS. The samples were loaded on top of an iodixanol gradient consisting of 3 ml 40% w/v, 3 ml 20% w/v, 3 ml 10% w/v and 2.5 ml 5% w/v iodixanol in a 13.2 ml ultraclear tube (Beckman Coulter). Exosome pellets obtained by ultracentrifugation from urine of CKD patients were resuspended with 200 μl of PBS and overlaid onto the top of the gradient. The samples were centrifuged at 100,000 × *g* (SW41 Ti rotor, Beckman Coulter) for 16 h at 4°C. Fractions of 1 ml were collected, their density was measured at 340 nm based on the absorbance values from the standard curve constructed by 5%, 10%, 20%, 40% and 60% iodixanol solutions to estimate the density of each fraction collected from samples. EV containing fractions (with a density of 1.11–1.16 g/ml) and the three closest fractions above and below the density 1.11–1.16 g/ml separately collected and used as control samples respectively were pooled and diluted with 30 ml in 1×PBS. Diluted samples were centrifuged at 160,000 × *g* (70 Ti rotor, Beckma Coulter) for 2 h at 4°C. The pellets were resuspended in 100 μl of PBS and stored at –80℃ until further analysis.

### 4.23 Quantifications of renal fibrosis

An Olympus DP80 microscope with an EMCCD camera was used to observe the glass slides stained with Masson stain under the microscope, and 2448 × 1920 pixel resolution images captured at a high magnification (×400) field from a randomly selected field were captured. Each part contains 10 fields, and the image of each part is divided into 100 squares. The tissue fibrosis stained in blue was scored.

### 4.24 Statistical analyses

All data are presented as mean ± SEM. Statistical analysis was performed using SPSS 19.0 (SPSS Inc, Chicago, IL). Comparisons were made by Student's *t*‐test for comparison of two groups, or via one‐way analysis of variance followed by the least significant difference or Games–Howell procedure for comparison of more than two groups. *p*‐values < 0.05 was considered statistically significant. Bivariate correlation analysis was performed using Pearson and Spearman rank correlation analysis.

## CONFLICT OF INTERESTS

The authors declare no competing financial interest.

## AUTHOR CONTRIBUTIONS

Shuangqin Chen, Meijia Zhang, Jiemei Li, Jiewu Huang, Shan Zhou, Xiaotao Hou, Huiyun Ye, Xi Liu, Weiwei Shen, Jinhua Miao, Fan Fan Hou, Youhua Liu, Shaowei Xiang and Lili Zhou conducted the experiments and prepared the materials involved in this study. Lili Zhou conceived this study. Lili Zhou participated in its design and coordination. Lili Zhou, Shuangqin Chen, Jiemei Li contributed to the analysis and interpretation of the data. Shuangqin Chen, Meijia Zhang, Jiewu Huang and Lili Zhou drafted the manuscript. All authors read and approved the final manuscript.

## Supporting information



Supporting Information

## Data Availability

Transcriptomic data produced in this study are available at NCBI with accession number GSE193282. The mass spectrometry proteomics data are available via ProteomeXchange with identifier PXD030696. The authors declare that the data supporting the findings of this study are available within the paper and its supplementary information file.

## References

[jev212203-bib-0001] Bai, G. , Matsuba, T. , Niki, T. , & Hattori, T. (2020). Stimulation of THP‐1 macrophages with LPS increased the production of osteopontin‐encapsulating exosome. International Journal of Molecular Sciences, 21(22), 8490. 10.3390/ijms21228490 PMC7696453 33187327

[jev212203-bib-0002] Cao, J.‐Y. , Wang, B. , Tang, T.‐T. , Wen, Y. , Li, Z.‐L. , Feng, S.‐T. , Wu, M. , Liu, D. , Yin, D. , Ma, K.‐L. , Tang, R.‐N. , Wu, Q.‐L. , Lan, H.‐Y. , Lv, L.‐L. , & Liu, B.‐C. (2021). Exosomal miR‐125b‐5p deriving from mesenchymal stem cells promotes tubular repair by suppression of p53 in ischemic acute kidney injury. Theranostics, 11(11), 5248–5266. 33859745 10.7150/thno.54550 PMC8039965

[jev212203-bib-0003] Chellaiah, M. A. , Biswas, R. S. , Rittling, S. R. , Denhardt, D. T. , & Hruska, K. A. (2003). Rho‐dependent Rho kinase activation increases CD44 surface expression and bone resorption in osteoclasts. Journal of Biological Chemistry, 278(31), 29086–29097. 10.1074/jbc.M211074200 12730217

[jev212203-bib-0004] Chen, C. , Zhao, S. , Karnad, A. , & Freeman, J. W. (2018). The biology and role of CD44 in cancer progression: Therapeutic implications. Journal of Hematology & Oncology, 11(1), 64. 29747682 10.1186/s13045-018-0605-5 PMC5946470

[jev212203-bib-0005] Conduit, S. E. , Hakim, S. , Feeney, S. J. , Ooms, L. M. , Dyson, J. M. , Abud, H. E. , & Mitchell, C. A. (2019). β‐catenin ablation exacerbates polycystic kidney disease progression. Human Molecular Genetics, 28(2), 230–244. 30265301 10.1093/hmg/ddy309

[jev212203-bib-0006] Feng, Y. , Liu, B. , Lee, K. , & He, J. C. (2021). A novel mechanism of regulation for exosome secretion in the diabetic kidney. Diabetes, 70(7), 1440–1442. 34155044 10.2337/dbi21-0015 PMC8336009

[jev212203-bib-0007] Gewin, L. S. (2018). Renal fibrosis: Primacy of the proximal tubule. Matrix Biology, 68‐69, 248–262. 10.1016/j.matbio.2018.02.006 PMC6015527 29425694

[jev212203-bib-0008] Gewin, L. , Zent, R. , & Pozzi, A. (2017). Progression of chronic kidney disease: Too much cellular talk causes damage. Kidney International, 91(3), 552–560. 27773427 10.1016/j.kint.2016.08.025 PMC5313325

[jev212203-bib-0009] Grande, M. T. , Sánchez‐Laorden, B. , López‐Blau, C. , De Frutos, C. A. , Boutet, A. , Arévalo, M. , Rowe, R. G. , Weiss, S. J. , López‐Novoa, J. M. , & Nieto, M. A. (2015). Snail1‐induced partial epithelial‐to‐mesenchymal transition drives renal fibrosis in mice and can be targeted to reverse established disease. Nature Medicine, 21(9), 989–997. 10.1038/nm.3901 26236989

[jev212203-bib-0010] Guan, H. , Peng, R. , Mao, L. , Fang, F. , Xu, B. , & Chen, M. (2020). Injured tubular epithelial cells activate fibroblasts to promote kidney fibrosis through miR‐150‐containing exosomes. Experimental Cell Research, 392(2), 112007. 32315664 10.1016/j.yexcr.2020.112007

[jev212203-bib-0011] Hattori, T. , Iwasaki‐Hozumi, H. , Bai, G. , Chagan‐Yasutan, H. , Shete, A. , Telan, E. F. , Takahashi, A. , Ashino, Y. , & Matsuba, T. (2021). Both full‐length and protease‐cleaved products of osteopontin are elevated in infectious diseases. Biomedicines, 9(8), 1006. 34440210 10.3390/biomedicines9081006 PMC8394573

[jev212203-bib-0012] He, C. , Zheng, S. , Luo, Y. , & Wang, B. (2018). Exosome theranostics: Biology and translational medicine. Theranostics, 8(1), 237–255. 29290805 10.7150/thno.21945 PMC5743472

[jev212203-bib-0013] He, L. , Wei, Q. , Liu, J. , Yi, M. , Liu, Y. , Liu, H. , Sun, L. , Peng, Y. , Liu, F. , Venkatachalam, M. A. , & Dong, Z. (2017). AKI on CKD: Heightened injury, suppressed repair, and the underlying mechanisms. Kidney International, 92(5), 1071–1083. 28890325 10.1016/j.kint.2017.06.030 PMC5683166

[jev212203-bib-0014] Hoac, B. , Susan‐Resiga, D. , Essalmani, R. , Marcinkiweicz, E. , Seidah, N. G. , & Mckee, M. D. (2018). Osteopontin as a novel substrate for the proprotein convertase 5/6 (PCSK5) in bone. Bone, 107, 45–55. 29126984 10.1016/j.bone.2017.11.002

[jev212203-bib-0015] Huang, J. , Kong, Y. , Xie, C. , & Zhou, L. (2021). Stem/progenitor cell in kidney: Characteristics, homing, coordination, and maintenance. Stem Cell Research & Therapy, 12(1), 197. 33743826 10.1186/s13287-021-02266-0 PMC7981824

[jev212203-bib-0016] Humphreys, B. D. (2018). Mechanisms of renal fibrosis. Annual Review of Physiology, 80, 309–326. 10.1146/annurev-physiol-022516-034227 29068765

[jev212203-bib-0017] Jha, V. , Wang, A. Y.‐M. , & Wang, H. (2012). The impact of CKD identification in large countries: The burden of illness. Nephrology, Dialysis, Transplantation, 27(Suppl 3), iii32–iii38. 10.1093/ndt/gfs113 23115140

[jev212203-bib-0018] Jing, H. , Tang, S. , Lin, S. , Liao, M. , Chen, H. , Fan, Y. , & Zhou, J. (2020). Adiponectin in renal fibrosis. Aging (Albany NY), 12(5), 4660–4672. PMID: 32065783. 32065783 10.18632/aging.102811 PMC7093169

[jev212203-bib-0019] Jing, H. , Tang, S. , Lin, S. , Liao, M. , Chen, H. , & Zhou, J. (2019). The role of extracellular vesicles in renal fibrosis. Cell Death & Disease, 10(5), 367. 31068572 10.1038/s41419-019-1605-2 PMC6506498

[jev212203-bib-0020] Kaleta, B. (2019). The role of osteopontin in kidney diseases. Inflammation Research, 68(2), 93–102. 30456594 10.1007/s00011-018-1200-5

[jev212203-bib-0021] Kang, H. M. , Ahn, S. H. , Choi, P. , Ko, Y.‐A. , Han, S. H. , Chinga, F. , Park, A. S. D. , Tao, J. , Sharma, K. , Pullman, J. , Bottinger, E. P. , Goldberg, I. J. , & Susztak, K. (2015). Defective fatty acid oxidation in renal tubular epithelial cells has a key role in kidney fibrosis development. Nature Medicine, 21(1), 37–46. 10.1038/nm.3762 PMC4444078 25419705

[jev212203-bib-0022] Klement, J. D. , Paschall, A. V. , Redd, P. S. , Ibrahim, M. L. , Lu, C. , Yang, D. , Celis, E. , Abrams, S. I. , Ozato, K. , & Liu, K. (2018). An osteopontin/CD44 immune checkpoint controls CD8+ T cell activation and tumor immune evasion. Journal of Clinical Investigation, 128(12), 5549–5560. 10.1172/JCI123360 PMC6264631 30395540

[jev212203-bib-0023] Krolikoski, M. , Monslow, J. , & Puré, E. (2019). The CD44‐HA axis and inflammation in atherosclerosis: A temporal perspective. Matrix Biology, 78–79, 201–218. 10.1016/j.matbio.2018.05.007 PMC6249119 29792915

[jev212203-bib-0024] Lee, M. N. , Song, J. H. , Oh, S.‐H. , Tham, N. T. , Kim, J.‐W. , Yang, J.‐W. , Kim, E.‐S. , & Koh, J.‐T. (2020). The primary cilium directs osteopontin‐induced migration of mesenchymal stem cells by regulating CD44 signaling and Cdc42 activation. Stem Cell Research, 45, 101799. 32339903 10.1016/j.scr.2020.101799

[jev212203-bib-0025] Li, Z.‐L. , Lv, L.‐L. , Tang, T.‐T. , Wang, B. , Feng, Y. , Zhou, L.‐T. , Cao, J.‐Y. , Tang, R.‐N. , Wu, M. , Liu, H. , Crowley, S. D. , & Liu, B.‐C. (2019). HIF‐1alpha inducing exosomal microRNA‐23a expression mediates the cross‐talk between tubular epithelial cells and macrophages in tubulointerstitial inflammation. Kidney International, 95(2), 388–404. 30551896 10.1016/j.kint.2018.09.013

[jev212203-bib-0026] Lin, R. , Wu, S. , Zhu, D. , Qin, M. , & Liu, X. (2020). Osteopontin induces atrial fibrosis by activating Akt/GSK‐3β/β‐catenin pathway and suppressing autophagy. Life Sciences, 245, 117328. 31954162 10.1016/j.lfs.2020.117328

[jev212203-bib-0027] Liu, H. , Zhang, Y. , Song, W. , Sun, Y. , & Jiang, Y. (2021). Osteopontin N‐terminal function in an abdominal aortic aneurysm from apolipoprotein E‐deficient mice. Frontiers in Cell and Developmental Biology, 9, 681790. 34458254 10.3389/fcell.2021.681790 PMC8397420

[jev212203-bib-0028] Liu, X. , Miao, J. , Wang, C. , Zhou, S. , Chen, S. , Ren, Q. , Hong, X. , Wang, Y. , Hou, F. F. , Zhou, L. , & Liu, Y. (2020). Tubule‐derived exosomes play a central role in fibroblast activation and kidney fibrosis. Kidney International, 97(6), 1181–1195. 32139089 10.1016/j.kint.2019.11.026

[jev212203-bib-0029] Liu, Y. (2011). Cellular and molecular mechanisms of renal fibrosis. Nature Reviews Nephrology, 7(12), 684–696. 22009250 10.1038/nrneph.2011.149 PMC4520424

[jev212203-bib-0030] Lv, L.‐L. , Feng, Y. , Wen, Y. , Wu, W.‐J. , Ni, H.‐F. , Li, Z.‐L. , Zhou, L.‐T. , Wang, B. , Zhang, J.‐D. , Crowley, S. D. , & Liu, B.‐C. (2018). Exosomal CCL2 from tubular epithelial cells is critical for albumin‐induced tubulointerstitial inflammation. Journal of the American Society of Nephrology, 29(3), 919–935. 29295871 10.1681/ASN.2017050523 PMC5827595

[jev212203-bib-0031] Lv, L.‐L. , Feng, Y. , Wu, M. , Wang, B. , Li, Z.‐L. , Zhong, X. , Wu, W.‐J. , Chen, J. , Ni, H.‐F. , Tang, T.‐T. , Tang, R.‐N. , Lan, H.‐Y. , & Liu, B.‐C. (2020). Exosomal miRNA‐19b‐3p of tubular epithelial cells promotes M1 macrophage activation in kidney injury. Cell Death and Differentiation, 27(1), 210–226. 31097789 10.1038/s41418-019-0349-y PMC7206053

[jev212203-bib-0032] Miao, J. , Liu, J. , Niu, J. , Zhang, Y. , Shen, W. , Luo, C. , Liu, Y. , Li, C. , Li, H. , Yang, P. , Liu, Y. , Hou, F. F. , & Zhou, L. (2019). Wnt/β‐catenin/RAS signaling mediates age‐related renal fibrosis and is associated with mitochondrial dysfunction. Aging Cell, 18(5), e13004. 31318148 10.1111/acel.13004 PMC6718575

[jev212203-bib-0033] Nastase, M. V. , Zeng‐Brouwers, J. , Wygrecka, M. , & Schaefer, L. (2018). Targeting renal fibrosis: Mechanisms and drug delivery systems. Advanced Drug Delivery Reviews, 129, 295–307. 29288033 10.1016/j.addr.2017.12.019

[jev212203-bib-0034] Pegtel, D. M. , & Gould, S. J. (2019). Exosomes. Annual Review of Biochemistry, 88, 487–514. 10.1146/annurev-biochem-013118-111902 31220978

[jev212203-bib-0035] Plantman, S. (2012). Osteopontin is upregulated after mechanical brain injury and stimulates neurite growth from hippocampal neurons through β1 integrin and CD44. Neuroreport, 23(11), 647–652. 22692550 10.1097/WNR.0b013e328355380e

[jev212203-bib-0036] Prunotto, M. , Budd, D. C. , Gabbiani, G. , Meier, M. , Formentini, I. , Hartmann, G. , Pomposiello, S. , & Moll, S. (2012). Epithelial‐mesenchymal crosstalk alteration in kidney fibrosis. Journal of Pathology, 228(2), 131–147. 10.1002/path.4049 22570261

[jev212203-bib-0037] Qi, R. , & Yang, C. (2018). Renal tubular epithelial cells: The neglected mediator of tubulointerstitial fibrosis after injury. Cell Death & Disease, 9(11), 1126. 30425237 10.1038/s41419-018-1157-x PMC6233178

[jev212203-bib-0038] Subraman, V. , Thiyagarajan, M. , Malathi, N. , & Rajan, S.‐T. (2015). OPN ‐Revisited. Journal of Clinical and Diagnostic Research, 9(6), ZE10–3. 10.7860/JCDR/2015/12872.6111 PMC4525627 26266236

[jev212203-bib-0039] Tan, R. J. , Zhou, D. , & Liu, Y. (2016). Signaling crosstalk between tubular epithelial cells and interstitial fibroblasts after kidney injury. Kidney Diseases (Basel), 2(3), 136–144. 10.1159/000446336 PMC5123005 27921041

[jev212203-bib-0040] Tang, T.‐T. , Wang, B. , Wu, M. , Li, Z.‐L. , Feng, Y. , Cao, J.‐Y. , Yin, D. , Liu, H. , Tang, R.‐N. , Crowley, S. D. , Lv, L.‐L. , & Liu, B.‐C. (2020). Extracellular vesicle‐encapsulated IL‐10 as novel nanotherapeutics against ischemic AKI. Science Advances, 6(33), eaaz0748. 32851154 10.1126/sciadv.aaz0748 PMC7423360

[jev212203-bib-0041] Vianello, E. , Kalousová, M. , Dozio, E. , Tacchini, L. , Zima, T. , & Corsi Romanelli, M. M. (2020). Osteopontin: The molecular bridge between fat and cardiac‐renal disorders. International Journal of Molecular Sciences, 21(15), 5568. 10.3390/ijms21155568 PMC7432729 32759639

[jev212203-bib-0042] Wang, B. , Yin, Q. , Han, Y.‐C. , Wu, M. , Li, Z.‐L. , Tu, Y. , Zhou, L.‐T. , Wei, Q. , Liu, H. , Tang, R.‐N. , Cao, J.‐Y. , Lv, L.‐L. , & Liu, B.‐C. (2020). Effect of hypoxia‐inducible factor‐prolyl hydroxylase inhibitors on anemia in patients with CKD: A meta‐analysis of randomized controlled trials including 2804 patients. Renal Failure, 42(1), 912–925. 32869703 10.1080/0886022X.2020.1811121 PMC7946011

[jev212203-bib-0043] Wortzel, I. , Dror, S. , Kenific, C. M. , & Lyden, D. (2019). Exosome‐mediated metastasis: Communication from a distance. Developmental Cell, 49(3), 347–360. 31063754 10.1016/j.devcel.2019.04.011

[jev212203-bib-0044] Xiao, H.‐B. , Lu, X.‐Y. , Sun, Z.‐L. , & Zhang, H.‐B. (2011). Kaempferol regulates OPN‐CD44 pathway to inhibit the atherogenesis of apolipoprotein E deficient mice. Toxicology and Applied Pharmacology, 257(3), 405–411. 22005275 10.1016/j.taap.2011.09.024

[jev212203-bib-0045] Xiao, L. , Zhou, D. , Tan, R. J. , Fu, H. , Zhou, L. , Hou, F. F. , & Liu, Y. (2016). Sustained Activation of Wnt/β‐Catenin Signaling Drives AKI to CKD Progression. Journal of the American Society of Nephrology, 27(6), 1727–1740. PMID: 26453613. 26453613 10.1681/ASN.2015040449 PMC4884114

[jev212203-bib-0046] Xu, J. , Zhou, L. , & Liu, Y. (2020). Cellular senescence in kidney fibrosis: Pathologic significance and therapeutic strategies. Frontiers in Pharmacology, 11, 601325. 33362554 10.3389/fphar.2020.601325 PMC7759549

[jev212203-bib-0047] Yang, L. , Besschetnova, T. Y. , Brooks, C. R. , Shah, J. V. , & Bonventre, J. V. (2010). Epithelial cell cycle arrest in G2/M mediates kidney fibrosis after injury. Nature Medicine, 16(5), 535–543, 1p following 143. 10.1038/nm.2144 PMC3928013 20436483

[jev212203-bib-0048] Yang, X. , Sun, J. , Xia, D. , Can, X. , Liu, L. , Zhang, J. , Xu, H. , Du, N. , Liu, W. , Shen, F. , Zhang, Z. , Sun, Y. , & Xi, X. (2017). Capn4 Enhances Osteopontin Expression through Activation of the Wnt/β‐Catenin Pathway to Promote Epithelial Ovarian Carcinoma Metastasis. Cellular Physiology and Biochemistry, 42(1), 185–197. PMID: 28535511. 28535511 10.1159/000477310

[jev212203-bib-0049] Yoo, K. H. , Thornhill, B. A. , Forbes, M. S. , Coleman, C. M. , Marcinko, E. S. , Liaw, L. , & Chevalier, R. L. (2006). Osteopontin regulates renal apoptosis and interstitial fibrosis in neonatal chronic unilateral ureteral obstruction. Kidney International, 70(10), 1735–1741. 17003824 10.1038/sj.ki.5000357

[jev212203-bib-0050] Zhou, D. , Li, Y. , Lin, L. , Zhou, L. , Igarashi, P. , & Liu, Y. (2012). Tubule‐specific ablation of endogenous β‐catenin aggravates acute kidney injury in mice. Kidney International, 82(5), 537–547. PMID: 22622501. 22622501 10.1038/ki.2012.173 PMC3425732

[jev212203-bib-0051] Zhou, D. , & Liu, Y. (2016). Renal fibrosis in 2015: Understanding the mechanisms of kidney fibrosis. Nature Reviews Nephrology, 12(2), 68–70. 10.1038/nrneph.2015.215 PMC4868356 26714578

[jev212203-bib-0052] Zhou, D. , Tan, R. J. , Zhou, L. , Li, Y. , & Liu, Y. (2013). Kidney tubular β‐catenin signaling controls interstitial fibroblast fate via epithelial‐mesenchymal communication. Science Reports, 3, 1878. 10.1038/srep01878 PMC3662012 23698793

[jev212203-bib-0053] Zhou, L. , Li, Y. , He, W. , Zhou, D. , Tan, R. J. , Nie, J. , Hou, F. F. , & Liu, Y. (2015). Mutual antagonism of Wilms' tumor 1 and β‐catenin dictates podocyte health and disease. Journal of the American Society of Nephrology, 26(3), 677–691. 25071087 10.1681/ASN.2013101067 PMC4341470

[jev212203-bib-0054] Zhou, S. , Wu, Q. , Lin, X. , Ling, X. , Miao, J. , Liu, X. , Hu, C. , Zhang, Y. , Jia, N. , Hou, F. F. , Liu, Y. , & Zhou, L. (2021). Cannabinoid receptor type 2 promotes kidney fibrosis through orchestrating β‐catenin signaling. Kidney International, 99(2), 364–381. 33152447 10.1016/j.kint.2020.09.025

[jev212203-bib-0055] Zhou, S. , Wu, Q. , Lin, X. , Ling, X. , Miao, J. , Liu, X. , Hu, C. , Zhang, Y. , Jia, N. , Hou, F. F. , Liu, Y. , & Zhou, L. (2021). Cannabinoid receptor type 2 promotes kidney fibrosis through orchestrating beta‐catenin signaling. Kidney International, 99(2), 364–381. 33152447 10.1016/j.kint.2020.09.025

