# Supplementary material for: β‐catenin‐controlled tubular cell‐derived exosomes play a key role in fibroblast activation via the OPN‐CD44 axis
Source: J Extracell Vesicles. 2022 Mar 21;11(3):e12203. doi: 10.1002/jev2.12203 (PMC8936047; doi:10.1002/jev2.12203)
Supplement: Supplementary file 1 — Supporting Information [file JEV2-11-e12203-s001.docx]

***Supplementary table S1. Parameter values of healthy subjects and CKD patients***

| **Healthy subjects** | | | |
| --- | --- | --- | --- |
| No. | Gender | Age | Urinary N-OPN(pmol/μmol) |
| 1 | M | 30 | 0.256462754 |
| 2 | F | 26 | 0.301067854 |
| 3 | F | 27 | 0.313724396 |
| 4 | F | 24 | 0.049453344 |
| 5 | F | 24 | 0.10557759 |
| 6 | M | 30 | 0.22617962 |
| 7 | F | 27 | 0.212230275 |
| 8 | F | 24 | 0.219877169 |
| 9 | F | 25 | 0.141549617 |
| 10 | F | 39 | 0.007592534 |
| 11 | M | 38 | 0.038729802 |
| 12 | F | 24 | 0.347088064 |
| 13 | M | 28 | 0.014312719 |
| 14 | M | 54 | 0.151169774 |
| 15 | M | 31 | 1.499892114 |
| 16 | M | 26 | 0.559982233 |
| 17 | F | 43 | 0.318294864 |
| 18 | F | 43 | 0.255823952 |
| 19 | M | 32 | 0.035772358 |
| 20 | M | 26 | 0.134109409 |
| 21 | M | 47 | 0.049309597 |
| 22 | M | 31 | 1.418512717 |
| 23 | M | 31 | 0.079963199 |
| 24 | M | 31 | 0.147248713 |
| 25 | M | 45 | 0.609924384 |
| 26 | F | 32 | 0.135977478 |
| 27 | F | 59 | 0.219218286 |
| 28 | F | 59 | 0.166845942 |
| 29 | F | 38 | 0.157181228 |
| 30 | F | 18 | 0.113224747 |

| **CKD patients** | | | | | |
| --- | --- | --- | --- | --- | --- |
| **No.** | **Gender** | **Age** | **Urinary N-OPN(pmol/μmol Ucr.)** | **eGFR(ml/min/1.73m^2^)** | **ACR(mg/mmol)** |
| 1 | F | 33 | 0.460809571 | 62 | 167.87 |
| 2 | F | 34 | 2.47619274 | 113 | 136.81 |
| 3 | F | 47 | 0.838607676 | 50 | 121.44 |
| 4 | F | 35 | 0.706975827 | 78 | 62.05 |
| 5 | F | 34 | 0.234926886 | 91 | 43.32 |
| 6 | F | 32 | 0.814030593 | 32 | 135.62 |
| 7 | F | 35 | 0.203605239 | 116 | 19.28 |
| 8 | M | 45 | 0.664269995 | 44 | 106.4 |
| 9 | M | 21 | 0.205879522 | 99 | 1.2 |
| 10 | F | 50 | 0.776135164 | 56 | 40.59 |
| 11 | F | 54 | 0.826415897 | 78 | 19.25 |
| 12 | M | 28 | 0.027484777 | 95 | 63.94 |
| 13 | F | 31 | 0.121845448 | 23 | 815.14 |
| 14 | M | 17 | 0.587560817 | 128 | 216.75 |
| 15 | F | 54 | 0.329921269 | 93 | 382.12 |
| 16 | M | 28 | 0.036341066 | 101 | 24.04 |
| 17 | F | 37 | 0.345222598 | 112 | 14.78 |
| 18 | F | 28 | 0.09572896 | 84 | 89.1 |
| 19 | M | 32 | 0.110215721 | 94 | 0.98 |
| 20 | M | 32 | 0.106567993 | 42 | 18.1 |
| 21 | F | 30 | 0.338842767 | 121 | 41.02 |
| 22 | F | 37 | 0.110904761 | 135 | 12.45 |
| 23 | F | 23 | 0.008361204 | 34 | 93.83 |
| 24 | M | 14 | 1.161422488 | 159 | 332.51 |
| 25 | M | 32 | 0.302351688 | 110 | 58.29 |
| 26 | M | 29 | 0.047310738 | 61 | 38.38 |
| 27 | M | 64 | 0.791140461 | 34 | 327.43 |
| 28 | M | 21 | 0.036516452 | 133 | 99.83 |
| 29 | M | 48 | 0.020107863 | 55 | 33.63 |
| 30 | F | 30 | 1.89062852 | 47 | 220.31 |
| 31 | M | 53 | 0.233966155 | 80 | 94.05 |
| 32 | M | 38 | 0.569083879 | 74 | 3.98 |
| 33 | M | 32 | 0.062209461 | 105 | 22.02 |
| 34 | M | 54 | 0.475597854 | 39 | 43.07 |
| 35 | M | 34 | 0.436287698 | 97 | 155.98 |
| 36 | M | 21 | 0.008836749 | 106 | 138.52 |
| 37 | F | 27 | 0.271921614 | 108 | 6.96 |
| 38 | F | 46 | 0.378548439 | 84 | 67.89 |
| 39 | M | 36 | 0.53574355 | 118 | 90.58 |
| 40 | F | 36 | 1.073957514 | 62 | 6.95 |
| 41 | F | 24 | 2.697599593 | 135 | 431.73 |
| 42 | F | 31 | 1.995912088 | 111 | 28.72 |
| 43 | F | 45 | 3.572663359 | 97 | 140.19 |
| 44 | M | 74 | 1.504680575 | 8 | 576.18 |
| 45 | M | 28 | 1.236823607 | 115 | 45.28 |
| 46 | M | 27 | 0.68497439 | 121 | 14.48 |
| 47 | M | 59 | 1.511732255 | 80 | 228.71 |
| 48 | M | 26 | 0.124108568 | 80 | 12.51 |
| 49 | F | 42 | 0.2169387 | 77 | 4.95 |
| 50 | F | 61 | 3.73965346 | 83 | 997.87 |
| 51 | M | 28 | 0.0720444 | 117 | 7 |
| 52 | F | 49 | 0.116467558 | 35 | 200.33 |
| 53 | F | 31 | 2.175022828 | 116 | 50.47 |
| 54 | M | 54 | 0.0533752 | 57 | 15.33 |
| 55 | M | 20 | 0.220957193 | 90 | 12.62 |
| 56 | F | 16 | 2.011692006 | 84 | 506.36 |
| 57 | F | 23 | 1.381044313 | 32 | 281.98 |
| 58 | F | 27 | 1.780062654 | 67 | 537.95 |
| 59 | M | 54 | 1.061867935 | 79 | 86.59 |
| 60 | M | 16 | 1.249185505 | 126 | 359.5 |
| 61 | M | 61 | 4.411440661 | 93 | 811.9 |
| 62 | M | 46 | 2.359423503 | 113 | 772.5 |
| 63 | M | 38 | 0.80046294 | 87 | 5.54 |
| 64 | F | 38 | 0.266793929 | 82 | 104.53 |
| 65 | F | 52 | 0.018785321 | 64 | 48.77 |
| 66 | M | 63 | 4.091744055 | 56 | 291.13 |
| 67 | F | 45 | 2.639783331 | 26 | 729.42 |
| 68 | F | 28 | 0.524429977 | 22 | 128.35 |
| 69 | M | 43 | 0.173143568 | 36 | 4.78 |
| 70 | F | 44 | 3.262997006 | 78 | 21.59 |
| 71 | M | 29 | 0.084594426 | 46 | 71.9 |
| 72 | F | 39 | 0.223067048 | 122 | 39.82 |
| 73 | M | 24 | 2.395323888 | 108 | 26.65 |
| 74 | F | 29 | 0.494034631 | 118 | 51.19 |
| 75 | M | 22 | 2.224656135 | 17 | 370.28 |
| 76 | F | 25 | 0.152500306 | 111 | 73.87 |
| 77 | F | 51 | 0.806699097 | 46 | 109.67 |
| 78 | M | 35 | 0.23534232 | 111 | 22.95 |
| 79 | M | 52 | 0.712059038 | 111 | 24.15 |
| 80 | M | 22 | 0.104113353 | 122 | 38.42 |
| 81 | M | 17 | 0.992228828 | 130 | 632.9 |
| 82 | M | 44 | 2.977810335 | 40 | 249.51 |
| 83 | F | 29 | 2.019752976 | 122 | 296.65 |
| 84 | M | 57 | 0.041171089 | 91 | 3.23 |
| 85 | F | 54 | 2.152904117 | 42 | 311.97 |
| 86 | M | 79 | 0.26198594 | 18 | 275.17 |
| 87 | M | 37 | 0.783709158 | 61 | 8.92 |
| 88 | F | 37 | 0.685534468 | 91 | 70.84 |
| 89 | F | 26 | 0.221402014 | 124 | 424.05 |
| 90 | F | 33 | 1.157066842 | 120 | 13.31 |
| 91 | M | 37 | 0.636349854 | 77 | 4.59 |
| 92 | F | 35 | 0.624588003 | 18 | 358.02 |
| 93 | M | 39 | 0.27168427 | 99 | 10.54 |
| 94 | F | 26 | 0.063671894 | 90 | 55.73 |
| 95 | M | 36 | 0.894099375 | 49 | 173.26 |
| 96 | M | 30 | 0.697914082 | 90 | 36.14 |
| 97 | F | 18 | 1.375697968 | 86 | 6.93 |
| 98 | F | 38 | 4.133413446 | 31 | 1521.27 |
| 99 | M | 13 | 0.7463366 | 117 | 556.47 |
| 100 | M | 43 | 2.391720107 | 110 | 300.5 |
| 101 | F | 41 | 0.055486116 | 94 | 123.92 |
| 102 | F | 83 | 1.322463768 | 37 | 760.25 |
| 103 | M | 84 | 0.403680475 | 35 | 6.17 |
| 104 | M | 64 | 0.326972536 | 35 | 67.59 |
| 105 | M | 63 | 1.322014284 | 34 | 501.68 |
| 106 | F | 54 | 0.222897314 | 50 | 38.54 |
| 107 | M | 37 | 0.096828225 | 32 | 5.26 |
| 108 | F | 58 | 0.549966239 | 56 | 318.96 |
| 109 | M | 85 | 3.040858574 | 34 | 343.84 |
| 110 | M | 22 | 1.041007481 | 55 | 37.27 |
| 111 | M | 56 | 1.281089003 | 44 | 1.34 |
| 112 | F | 45 | 0.68959088 | 40 | 17.32 |
| 113 | M | 67 | 5.478368243 | 40 | 117.49 |
| 114 | M | 91 | 0.799255272 | 41 | 144.18 |
| 115 | M | 69 | 2.821705426 | 34 | 102.3 |
| 116 | M | 85 | 0.398328188 | 28.93 | 10.61 |
| 117 | F | 26 | 3.01309078 | 26 | 463.17 |
| 118 | M | 48 | 0.191009778 | 16 | 210.35 |
| 119 | M | 74 | 0.727095938 | 18 | 4.11 |
| 120 | F | 39 | 0.130970367 | 24 | 157.49 |
| 121 | M | 53 | 1.58536931 | 20 | 420.39 |
| 122 | F | 74 | 0.203659447 | 19 | 16.98 |
| 123 | M | 52 | 0.462340539 | 27 | 499.94 |
| 124 | M | 86 | 0.396849772 | 24 | 34.58 |
| 125 | F | 47 | 0.714502033 | 28.86 | 20.08 |
| 126 | M | 36 | 1.848831548 | 28 | 174.54 |
| 127 | M | 58 | 1.16465783 | 9 | 308.95 |
| 128 | M | 70 | 0.179146126 | 27 | 7.48 |
| 129 | M | 30 | 1.22338403 | 29 | 286.71 |
| 130 | F | 69 | 0.487640489 | 21 | 10.96 |
| 131 | M | 60 | 4.563873627 | 25 | 696.77 |
| 132 | F | 79 | 0.826902553 | 26 | 65.5 |
| 133 | M | 65 | 0.201309329 | 19 | 73.26 |
| 134 | M | 77 | 0.181984892 | 15 | 8.15 |
| 135 | M | 84 | 1.906477198 | 29 | 526.48 |
| 136 | M | 53 | 0.085470085 | 30 | 150.61 |
| 137 | F | 50 | 3.707520549 | 20 | 132.43 |
| 138 | M | 70 | 0.21685761 | 28 | 88.36 |
| 139 | M | 72 | 0.217360276 | 23 | 170.1 |
| 140 | M | 85 | 0.319420427 | 8 | 92.57 |
| 141 | M | 61 | 1.412217272 | 15 | 464.15 |
| 142 | F | 71 | 1.512724659 | 9 | 275.2 |
| 143 | F | 52 | 2.178089926 | 5 | 766.32 |
| 144 | F | 75 | 3.062743708 | 12 | 11.99 |
| 145 | M | 67 | 0.941180964 | 3 | 4.05 |
| 146 | F | 34 | 1.048930449 | 6 | 117.29 |
| 147 | M | 62 | 0.81898962 | 2 | 1024.57 |
| 148 | F | 63 | 0.725729443 | 5 | 150.49 |
| 149 | F | 68 | 0.238250986 | 4 | 8.25 |
| 150 | M | 85 | 0.272538575 | 8 | 162.06 |
| 151 | M | 75 | 1.053007589 | 11 | 136.77 |
| 152 | M | 37 | 0.194719074 | 8 | 12.51 |
| 153 | M | 58 | 2.772021395 | 6 | 445.15 |
| 154 | F | 56 | 0.201827875 | 9 | 49.77 |
| 155 | F | 77 | 0.380778318 | 11 | 1006.68 |
| 156 | M | 55 | 0.215955637 | 8 | 89.29 |
| 157 | M | 47 | 2.287628048 | 9 | 188.92 |
| 158 | F | 69 | 2.384359316 | 9 | 210.12 |
| 159 | M | 54 | 0.583170292 | 11 | 361.41 |
| 160 | M | 60 | 3.082402731 | 10.25 | 1135.35 |
| 161 | M | 70 | 1.635096012 | 12 | 5.93 |
| 162 | M | 57 | 0.433510014 | 4 | 64.81 |
| 163 | M | 28 | 4.857932238 | 6 | 672.71 |
| 164 | F | 45 | 2.012871387 | 7 | 307.65 |
| 165 | M | 40 | 0.7238802 | 3 | 201.45 |
| 166 | F | 56 | 0.445685536 | 3 | 254.24 |
| 167 | M | 77 | 0.158032757 | 12 | 17.32 |
| 168 | F | 20 | 1.887021482 | 4 | 178.2 |
| 169 | F | 37 | 2.175624241 | 4 | 280.91 |
| 170 | M | 60 | 0.173950314 | 15 | 50.06 |
| 171 | M | 65 | 3.001238475 | 11.92 | 304.35 |
| 172 | M | 77 | 3.284573289 | 12 | 424.5 |
| 173 | M | 66 | 3.674211507 | 3 | 487.47 |
| 174 | F | 47 | 0.782492116 | 12 | 160.18 |
| 175 | M | 48 | 1.721100129 | 2 | 513.67 |
| 176 | M | 70 | 0.231947013 | 14 | 172.88 |
| 177 | F | 79 | 2.468242767 | 5 | 470.8 |
| 178 | F | 59 | 1.198756728 | 2 | 427.17 |
| 179 | M | 51 | 0.823013086 | 9 | 172.42 |
| 180 | F | 59 | 2.028517824 | 14 | 750.82 |
| 181 | M | 82 | 0.641554242 | 9.94 | 80.16 |
| 182 | M | 54 | 1.174092004 | 4 | 180.28 |
| 183 | F | 65 | 0.2989 | 9 | 566.4200 |

GFR: Glomerular filtration rate (ml/min/1.73m2); UACR: urine albumin-creatinine ratio (mg/mmol).

***Supplementary table S2. Nucleotide sequence of the primer used for qRT-PCR***

| Primer Sequence (5' to 3')  Gene Forward Reverse |
| --- |
| Wnt1 5'-CACCTCTTCGGCAAGATCGT-3' 5'-GTAGTCGCAGGTGCAGGATT-3'  Wnt3 5'-GTAGGGAAGCCAGGGCACAG-3' 5'-TGAGAAAGAAGCCGCCCATC-3'  Wnt4 5'-TTTGCAGTGACAAGAGCATGC-3' 5'-GAGCATCCTGACCACTGGAAA-3'  Wnt5a 5'-GCGGGACTTTCTCAAGGACA-3' 5'-CGGCTGCCTATTTGCATCAC-3'  Wnt5b 5'-AGCACCAGTTTCGACAGAGG-3' 5'-TGATGGCATTCACCACACCA-3'  Wnt6 5'-GGTCACTCAGGCCTGTTCC-3' 5'-TCGACATCATCTCCGCAGC-3  Wnt9a 5'-AAGCGAGGCTTCAAGGAGAC-3' 5'-TTTTCCAGGTCAGGTGCCTC-3  Wnt9b 5'-CAACCTGCAAGTGTCATGGC-3' 5'-CCAAGGCCTCATTGGTAGCA-3'  Wnt10b 5'-GGGTGGCTGTAATCACGACA-3' 5'-GCATTTGCACTTCCGCTTCA-3'  Wnt16 5'-TGGGCATTCAGGAGTGCAG-3' 5'-ATGCTGTCTCCTTGGTGCC-3'  CD44 5'-TCCACCATTGAGAAGAGCACC-3' 5'-CTGAGCTGTTGCATGGCTTTT-3'  SPP1 5'-GGCTTACGGACTGAGGTCAAA-3' 5'-TGACTTGACTCATGGCTGGTC-3'  β-actin 5'-AAGATCAAGATCATTGCTCCTCCTG-3' 5'-CGCAGCTCAGTAACAGTCCG-3' |


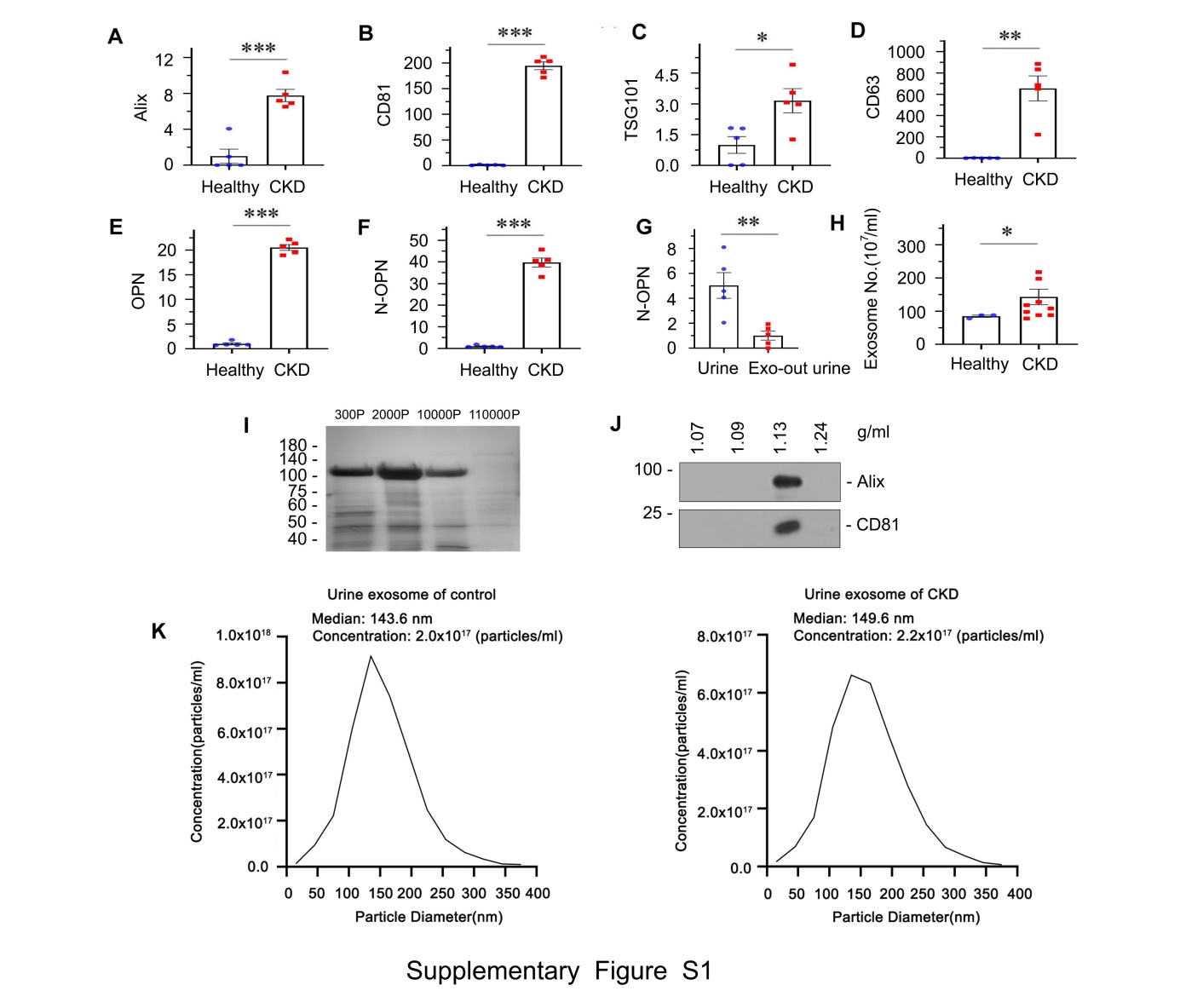


***Supplementary Figure S1. N-OPN and OPN were detected in exosome, which were identified no contaminant protein***

**(A–F)** Quantitative data on Alix **(A)**, CD81 **(B)**, TSG101 **(C)**, CD63 **(D)**, OPN **(E)**, and N-OPN **(F)** are shown. **P* < 0.05, ***P* < 0.01, ****P* < 0.001 versus Healthy; (n = 5). **(G)** Quantitative data for N-OPN are shown. ***P* < 0.01 versus urine (n = 5). **(H)** Quantification for exosome number is shown. **P* < 0.05 versus Healthy (n = 3 for healthy; n = 9 for CKD). **(I)** Representative gel analysis using silver staining of exosomes from the urine of CKD subjects. The precipitations (P) of urine from differential centrifugation were analyzed by silver staining. There is no THP protein (~110kDa) contamination in the pellets (exosomes) of 110,000g. **(J)** Exosome was purified from OptiPrep density gradient centrifugation to identify its purity. **(K)** Urinary exosome size and number were detected by NTA.

**
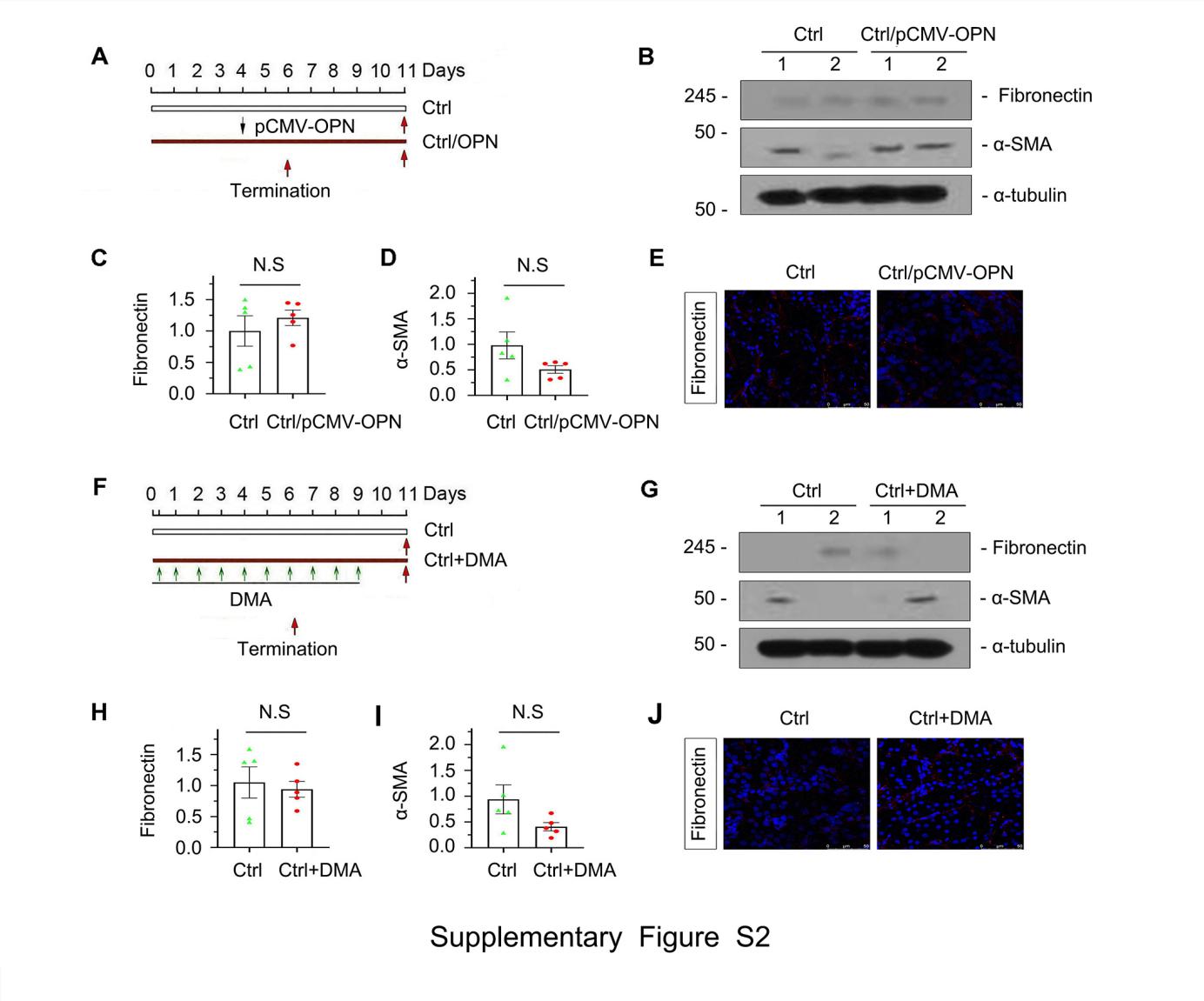
**

***Supplementary Figure S2. Overexpression of OPN or injection of DMA in normal mice does not aggravate renal fibrosis***

**(A)** Experimental design. Red arrows indicate the time points undergoing sacrifice. Black arrow indicates the injection of OPN expression plasmid (pCMV-OPN). **(B–D)**Western blot analyses showing that the expression of Fibronectin and α-SMA protein was not increased in two groups, as indicated. Representative western blot **(B)** and quantitative data of Fibronectin **(C)** and α-SMA **(D)** are shown. Numbers (1 to 2) indicate individual animal in a given group. N.S., not significant (n = 5). **(E)** Representative micrographs showing immunofluorescence staining of fibronectin in two groups, as indicated. scale bar: 50 µm. **(F)** Experimental design. Red arrows indicate the time points undergoing sacrifice. Green arrows indicate dimethyl amiloride (DMA) treatments (10 mg/kg body weight). **(G–I)**Representative Western blot **(G)** and quantitative data of Fibronectin **(H)** and α-SMA **(I)** are shown. N.S., not significant (n = 5). **(J)** Representative micrographs show immunofluorescence staining of fibronectin in two groups, as indicated. scale bar: 50 µm.

***
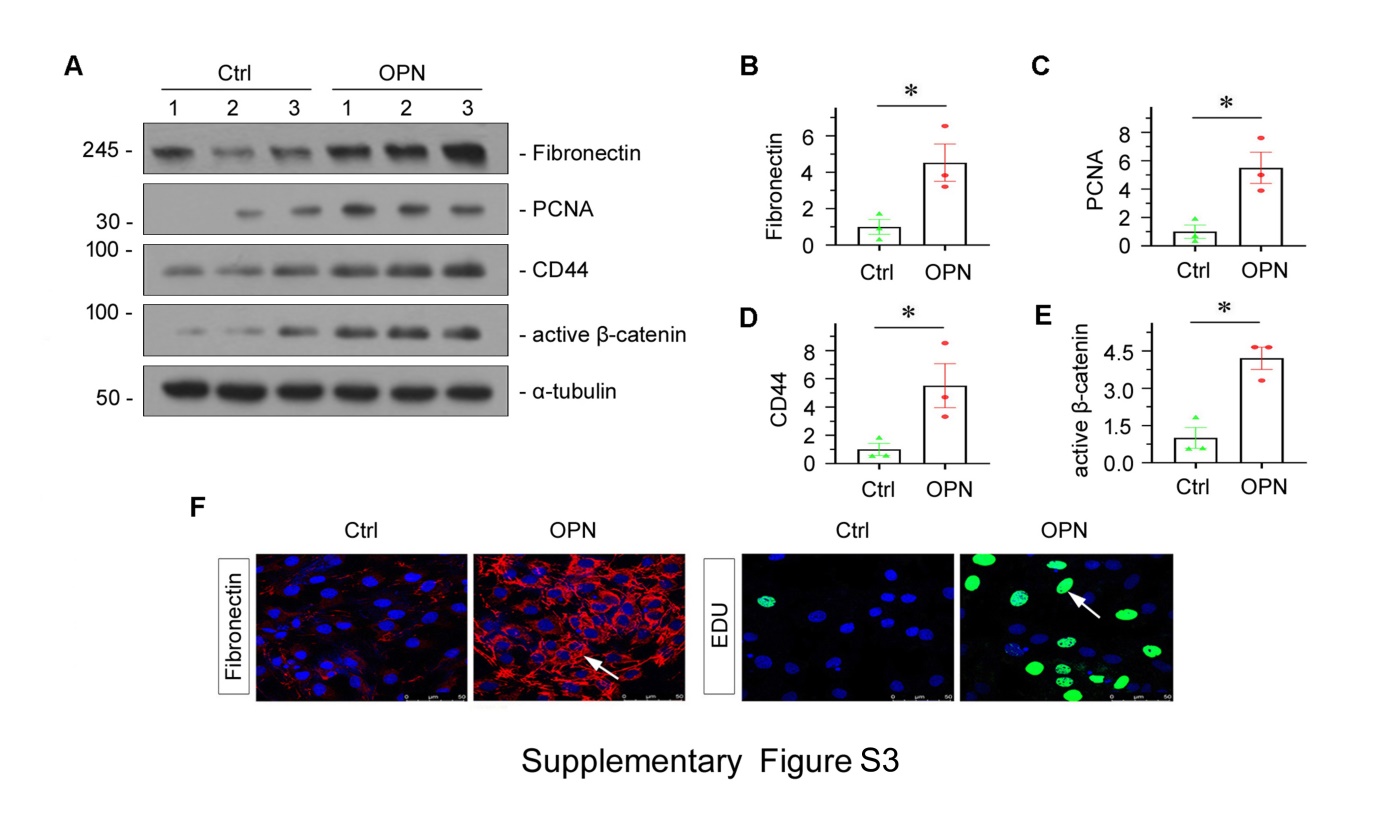
***

***Supplementary Figure S3.OPN promotes fibroblast activation in vitro***

NRK-49F cells were treated with 50 ng/ml of recombinant OPN for 24 h. **(A–E)** Western blot analyses showing the expression of Fibronectin, PCNA, CD44 and active β-catenin in NRK-49Fcells in two groups, as indicated. Representative western blot **(A)** and quantitative data of fibronectin **(B)**, PCNA **(C)**, CD44 **(D)** and active β-catenin **(E)** are shown. Numbers (1 to 3) indicate each individual treatment in a given group. **P* < 0.05 versus controls (n = 3). **(F)** Representative micrographs of immunofluorescence staining of Fibronectin and EDU in two groups, as indicated. Arrow indicates positive staining; Scale bar: 50 µm

***
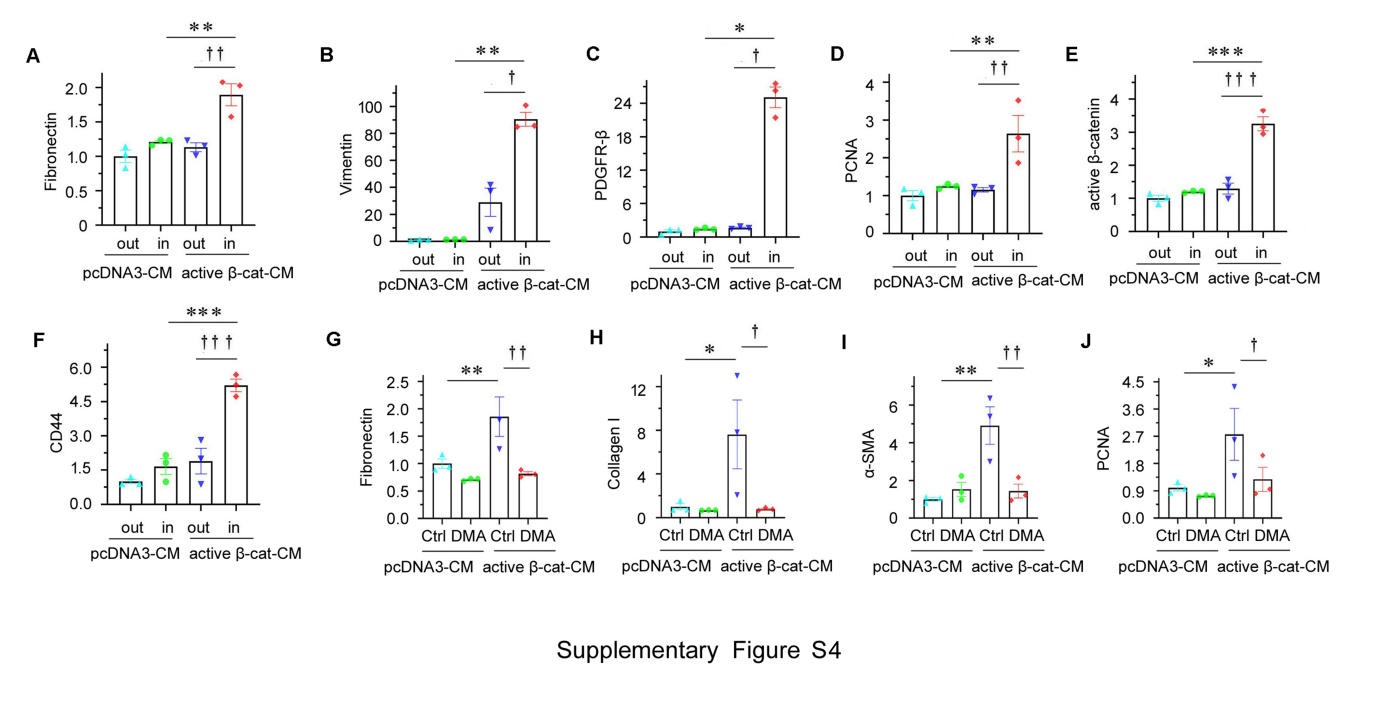
***

***Supplementary Figure S4. Tubule-derived exosomes play a major role in mediating fibroblast activation but DMA inhibited these effects in vitro***

**(A–F)** Quantitative data of fibronectin **(A)**, vimentin **(B)**, PDGFR-β **(C)**, PCNA **(D)**, active β-catenin **(E)**, and CD44 **(F)** are shown. **P* < 0.05, ***P* < 0.01, ****P* < 0.001 versus pcDNA3-CM with Exo in; **^†^***P* < 0.05, **^††^***P* < 0.01, **^†††^***P* < 0.001 versus active β-catenin-CM without Exo; (n = 3). **(G–J) Q**uantitative data of fibronectin **(G)**, collagen I **(H)**, α-SMA **(I)**, and PCNA **(J)** are shown. **P* < 0.05, ***P* < 0.01 versus pcDNA3-CM; **^†^***P* < 0.05, **^††^***P* < 0.01 versus active β-catenin-CM; (n = 3)


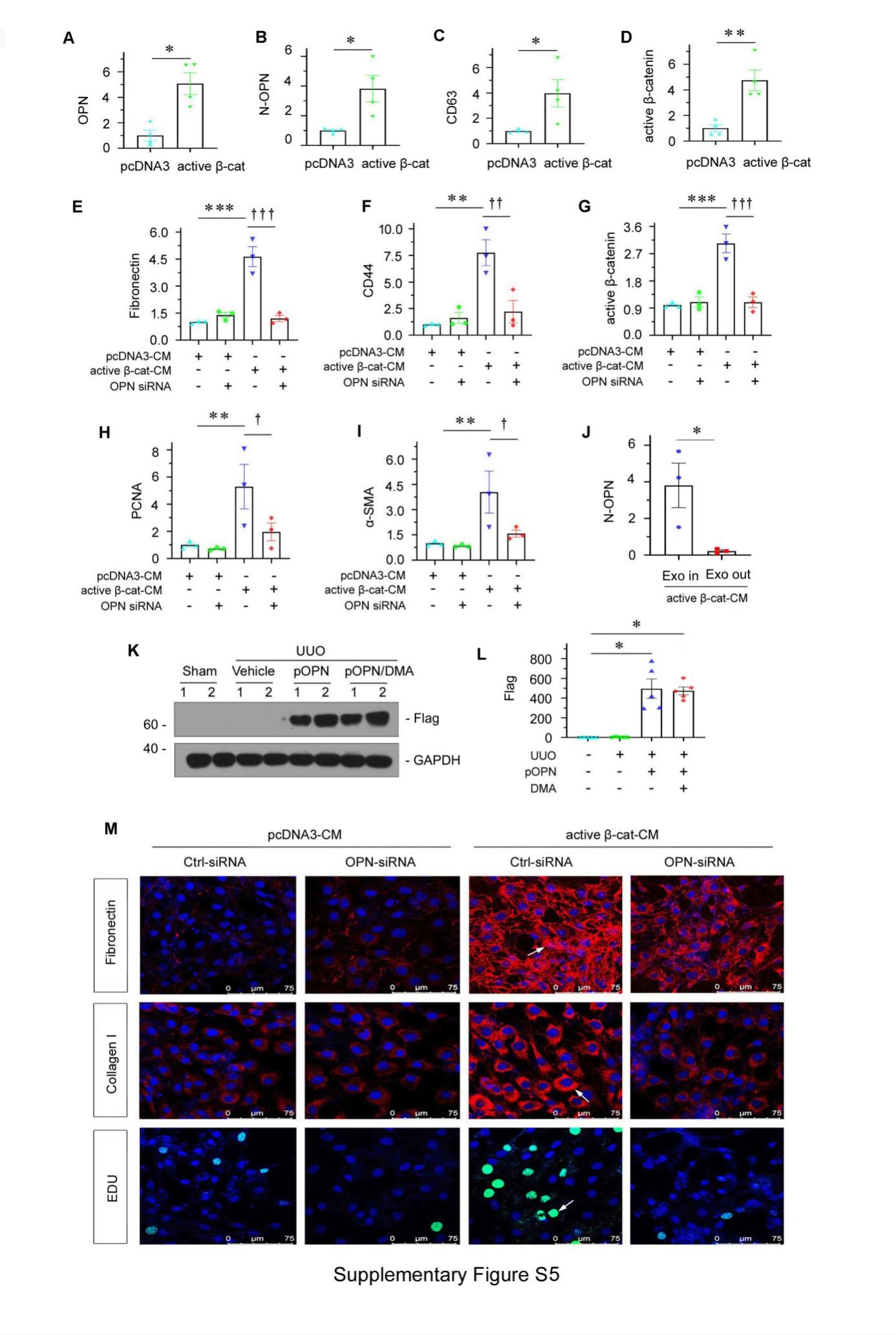


***Supplementary Figure S5. OPN encapsuled in tubule-derived exosomes plays a major role in mediating fibroblast activation***

**(A–D)** Western blot analyses show the expression of OPN, N-OPN, CD63 and active β-catenin in HKC-8 cells transfected with pDel-β-catenin. Quantitative data of OPN **(A)**, N-OPN **(B)**, CD63 **(C)** and active β-catenin **(D)** are shown. **P* < 0.05, ***P* < 0.01 versus pcDNA3 (n = 4). **(E–I)** Western blot analyses show that knockdown of OPN in HKC-8 cells abolished fibroblast activation in NRK-49F cells after incubation with conditioned media. Quantitative data of Fibronectin **(E)**, CD44 **(F)**, active β-catenin **(G)**, PCNA **(H)** and α-SMA **(I)** are shown. ***P* < 0.01, ****P* < 0.001 versus pcDNA3-CM; **^†^***P* < 0.05, **^††^***P* < 0.01, **^†††^***P* < 0.001 versus active β-catenin-CM (n = 3). **(J)** Quantitative data of N-OPN protein expression in supernatant and exosome-removed supernatant in HKC-8 cells transfected with pDel-β-catenin are shown. **P* < 0.05 versus Exo in group (n = 3). **(K–L)** Western blot analyses showing the expression of Flag in different groups, as indicated. Representative western blot **(K)** and quantitative data of Flag **(L)** are shown. Numbers (1 to 2) indicate each individual treatment in a given group. **P* < 0.05 versus controls (n = 5). **(M)** Representative micrographs of immunofluorescence staining of fibronectin, collagen Ⅰ, and EDU in different groups, as indicated; HKC-8 cells were co-transfected with siRNA to OPN and pDel-β-catenin plasmid (or pcDNA3), and supernatants were collect as active-β-catenin-CM or pcDNA3-CM. NRK-49F cells were treated with 40% of active-β-catenin-CM or pcDNA3-CM for 24 h. Scale bar: 75 µm.

***
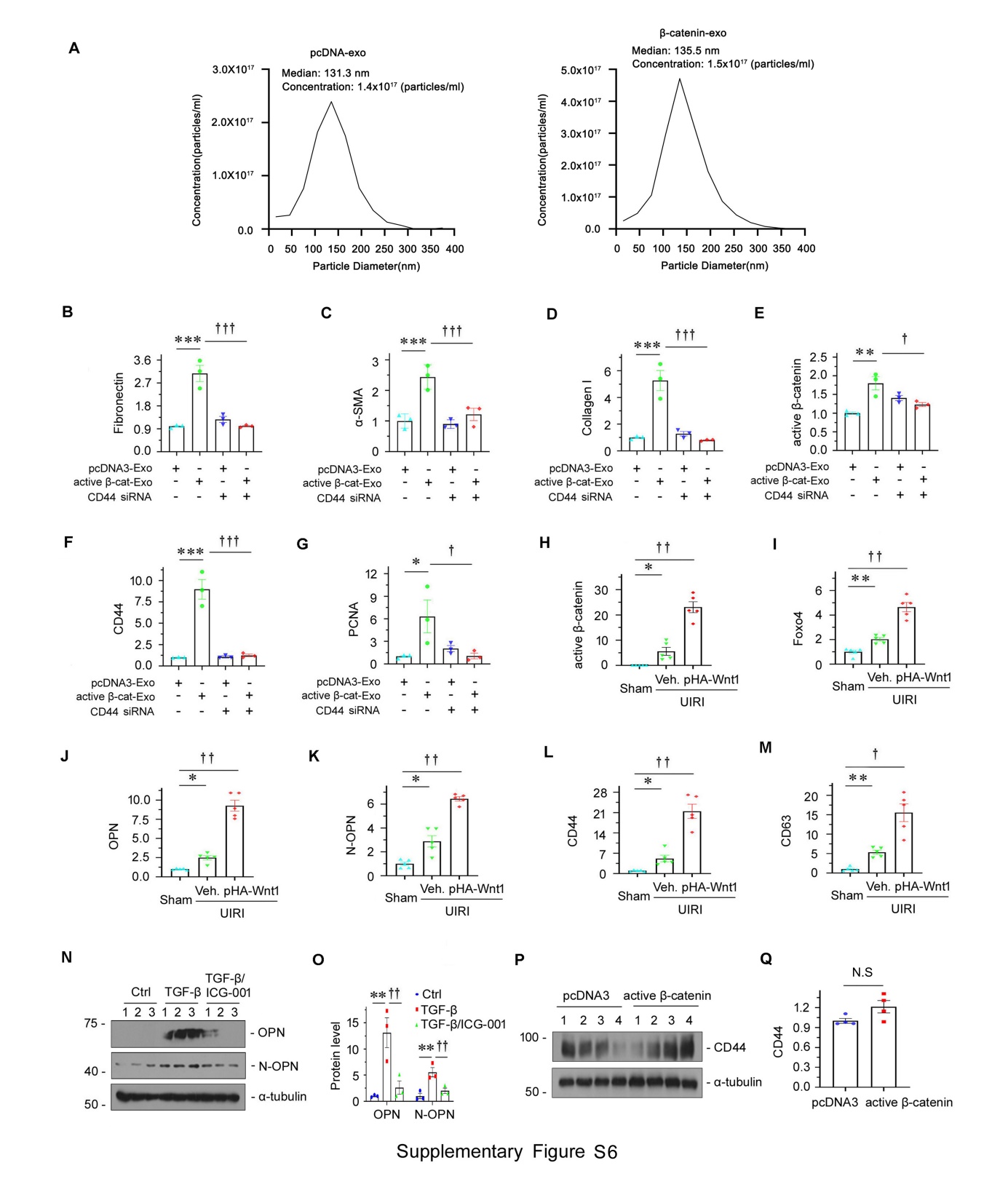
***

***Supplementary Figure S6. Exosome-mediated activation of the OPN/CD44 axis in tubule-fibroblast communication is controlled by β-catenin***

1. Exosomes were isolated from supernatant of HKC-8 cells transfected with pDel-β-catenin or pcDNA3. The size and numbers were detected by NTA. **(B–G)** Western blot analyses show that knockdown of CD44 in NRK-49F cells abolished fibroblast activation after incubation with tubule-derived exosomes (active β-catenin-Exo). Quantitative data on fibronectin **(B)**, α-SMA **(C)**, collagen I **(D)**, active β-catenin **(E)**, CD44 **(F)** and PCNA **(G)** are shown. **P* < 0.05, ***P* < 0.01, ****P* < 0.001 versus pcDNA3-Exo; **^†^***P* < 0.05, **^†††^***P* < 0.001 versus active β-catenin-Exo (n = 3). **(H–M)** Western blot analyses show that overexpression of Wnt1 in UIRI mice enhanced exosomal OPN/CD44 signal axis. Quantitative data of active β-catenin **(H)**, Foxo4 **(I)**, OPN **(J)**, N-OPN **(K)**, CD44 **(L)**, and CD63 **(M)** are shown. **P* < 0.05, ***P* < 0.01 versus sham controls; **^†^***P* < 0.05, **^††^***P* < 0.01 versus UIRI + pcDNA3 (Veh.) (n = 5). **(N–O)** ICG-001, a specific inhibitor of β-catenin, repressed the expression of OPN and N-OPN induced by TGF-β in HKC-8 cells. HKC-8 cells were pretreated with ICG-001 (5μM) for 1 h, and then treated with 2 ng/ml of TGF-β for 24 h. Representative western blot **(N)** and quantitative data of OPN and N-OPN **(O)** are shown. ***P* < 0.01, versus controls; **^††^***P* < 0.01 versus TGF-β (n = 3). **(P–Q)** Representative Western blot **(P)** and quantitative data of CD44 **(Q)** are shown. HKC-8 cells were transfected with pDel-β-catenin plasmid for 24 h. N.S., not significant (n = 4).


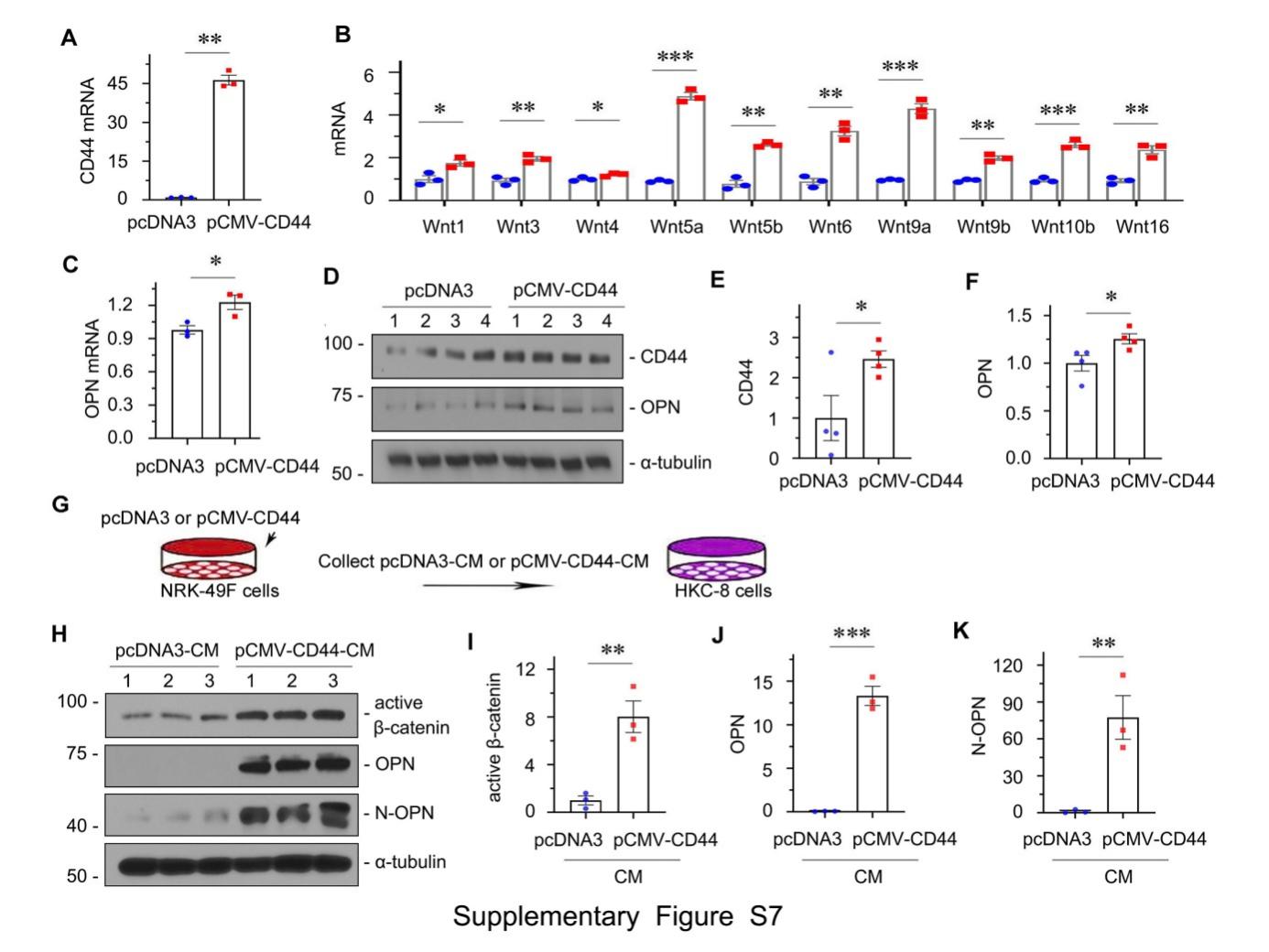


***Supplementary Figure S7. CD44 promotes up-regulation of multiple Wnts in fibroblasts and induced expression of β-catenin in tubular cells***

NRK-49F cells were transfected with CD44 expression plasmid or pcDNA3 for 24 h. (A–C) Quantitative real-time PCR analyses showing mRNA expression of CD44 (A), Wnt1, Wnt3, Wnt4, Wnt5a, Wnt5b, Wnt6, Wnt9a, Wnt9b, Wnt10b, Wnt16 (B) and OPN (C) in two groups, as indicated. **P* < 0.05, ***P* < 0.01, ****P* < 0.001 versus pcDNA3 (n = 3). (D–F) NRK-49F cells were transfected with CD44 expression plasmid for 24 h. Western blot analyses showing protein expression of CD44 and OPN in two groups, as indicated. Representative western blot (D) and quantitative data of CD44 (E) and OPN (F) are presented. **P* < 0.05 versus pcDNA3 (n = 4). (G) NRK-49F cells were transfected with CD44 expression plasmid (or pcDNA3) for 6h, washed with sterile PBS, and then incubated for an additional 24 h in serum-free medium to collect conditioned media (CM). HKC-8 cells were incubated with 40% of pCMV-CD44-CM (or pcDNA3-CM) for 24 hours. (H–K) Western blot analyses showing protein expression of active β-catenin, OPN and N-OPN in two groups, as indicated. Representative western blot (H) and quantitative data of active β-catenin (I), OPN (J) and N-OPN (K) are shown. ***P* < 0.01, ****P* < 0.001 versus pcDNA3-CM (n = 3).
